# Supplementary material for: Pollinator sharing, copollination, and speciation by host shifting among six closely related dioecious fig species
Source: Commun Biol. 2022 Apr 8;5:284. doi: 10.1038/s42003-022-03223-0 (PMC8993897; doi:10.1038/s42003-022-03223-0)
Supplement: Supplementary file 2 — Supplementary Information [file 42003_2022_3223_MOESM2_ESM.pdf]

Supplementary figures and tables

Pollinator sharing, copollination, and speciation by host shifting among six closely related dioecious fig species

Zhi-Hui Su[1,2], Ayako Sasaki[1], Junko Kusumi[3], Po-An Chou[4], Hsy-Yu Tzeng[4], Hong-Qing Li[5], & Hui Yu[6]

[1]JT Biohistory Research Hall, Takatsuki, Osaka 569-1125, Japan. [2]Department of Biological Sciences, Graduate School of Science, Osaka University, Osaka 560-0043, Japan. [3]Department of Environmental Changes, Faculty of Social and Cultural Studies, Kyushu University, Fukuoka 819-0395, Japan. [4]Department of Forestry, National Chung Hsing University, Taichung 402, Taiwan. [5]School of Life Sciences, East China Normal University, Shanghai 200241, China. [6]Guangdong Provincial Key Laboratory of Digital Botanical Garden, South China Botanical Garden, The Chinese Academy of Sciences, Guangzhou 510650, China.

Correspondence and requests for materials should be addressed to Z.-H. S. (emails: su.zhihui@brh.co.jp).

This file includes:

Supplementary Figure 1. BI tree of fig species based on 2198 SNPs from 80138 loci of MIG-seq data.

Supplementary Figure 2. Statistical-parsimony network of *F. erecta*, *F. formosana* and *F. vaccinioides* based on six non-coding regions (3715 sites) of the plastid DNA sequences.

Supplementary Figure 3. ML tree of ITS sequence (671 bp) from leaves and pollen (red and green, respectively) of four fig species.

Supplementary Figure 4. ML tree of pollinator wasps based on *COI-COII* sequences.

Supplementary Figure 5. BI tree of pollinator wasps based on *COI-COII* sequences.

Supplementary Figure 6. Sample localities of the pollinator wasps, *Blastophaga yeni* and *B*. sp. associated with *F. vaccinioides*.

Supplementary Figure 7. ML tree of pollinator wasps inferred by nuclear 28S rDNA sequences.

Supplementary Figure 8. ML tree of pollinator wasps based on ITS1 sequences.

Supplementary Figure 9. Species delimitation.

Supplementary Figure 10. The topology and chronogram based on 28S rDNA and *COI-COII* sequences of pollinator wasps.

Supplementary Figure 11. Population genetic structure of figs based on SSR data.

Supplementary Figure 12. Cophylogenetic analysis between figs and pollinator wasps.

Supplementary Table 1. Pairwise F_{ST} values between fig populations analyzed based on SSR data using Arlequin v3.5[68].

Supplementary Table 2. Pairwise F_{ST} values between pollinator wasp populations analyzed based on SSR data using Arlequin v3.5[68].

Supplementary Table 3. Primers used for PCR amplification in this study.

Supplementary Table 4. PCR amplification conditions.

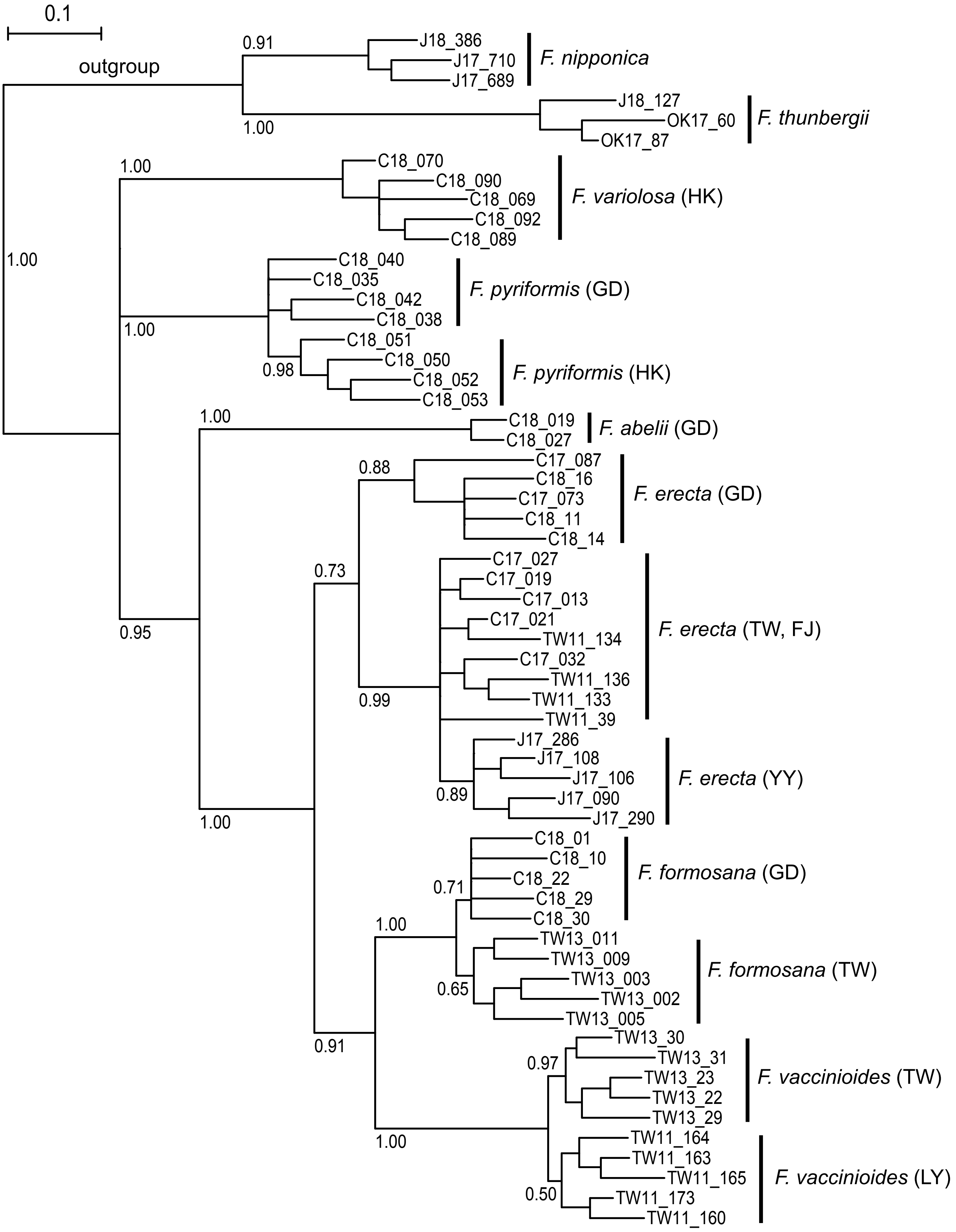

Supplementary Figure 1. BI tree of fig species based on 2198 SNPs from 80138 loci of MIG-seq data. The numbers at the nodes show posterior probability supports. For sample information, refer to Table 1 and Supplementary Data 1. For the abbreviations of population names in parentheses after the fig scientific names, refer to Fig. 1.

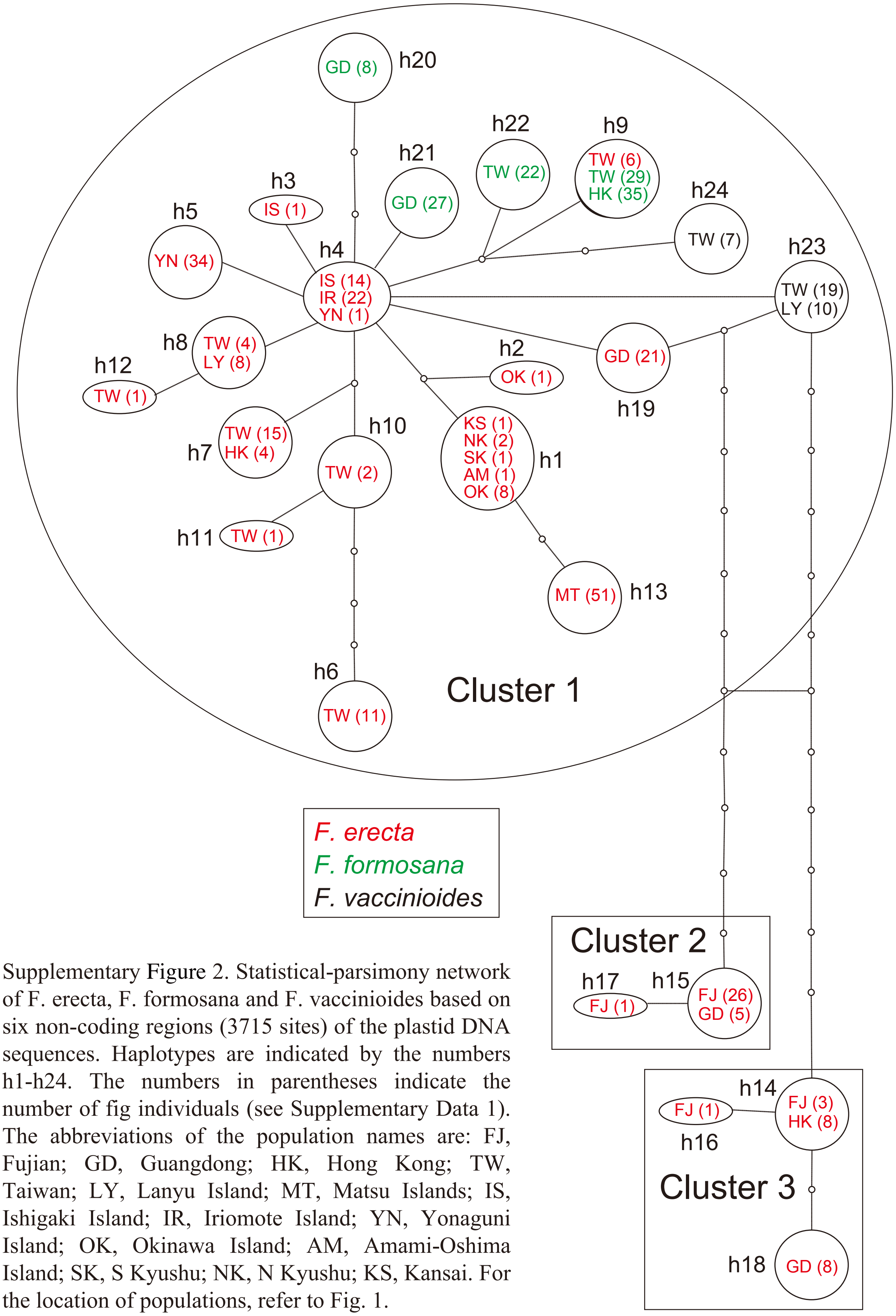

Supplementary Figure 2. Statistical-parsimony network of F. erecta, F. formosana and F. vaccinioides based on six non-coding regions (3715 sites) of the plastid DNA sequences. Haplotypes are indicated by the numbers h1-h24. The numbers in parentheses indicate the number of fig individuals (see Supplementary Data 1). The abbreviations of the population names are: FJ, Fujian; GD, Guangdong; HK, Hong Kong; TW, Taiwan; LY, Lanyu Island; MT, Matsu Islands; IS, Ishigaki Island; IR, Iriomote Island; YN, Yonaguni Island; OK, Okinawa Island; AM, Amami-Oshima Island; SK, S Kyushu; NK, N Kyushu; KS, Kansai. For the location of populations, refer to Fig. 1.

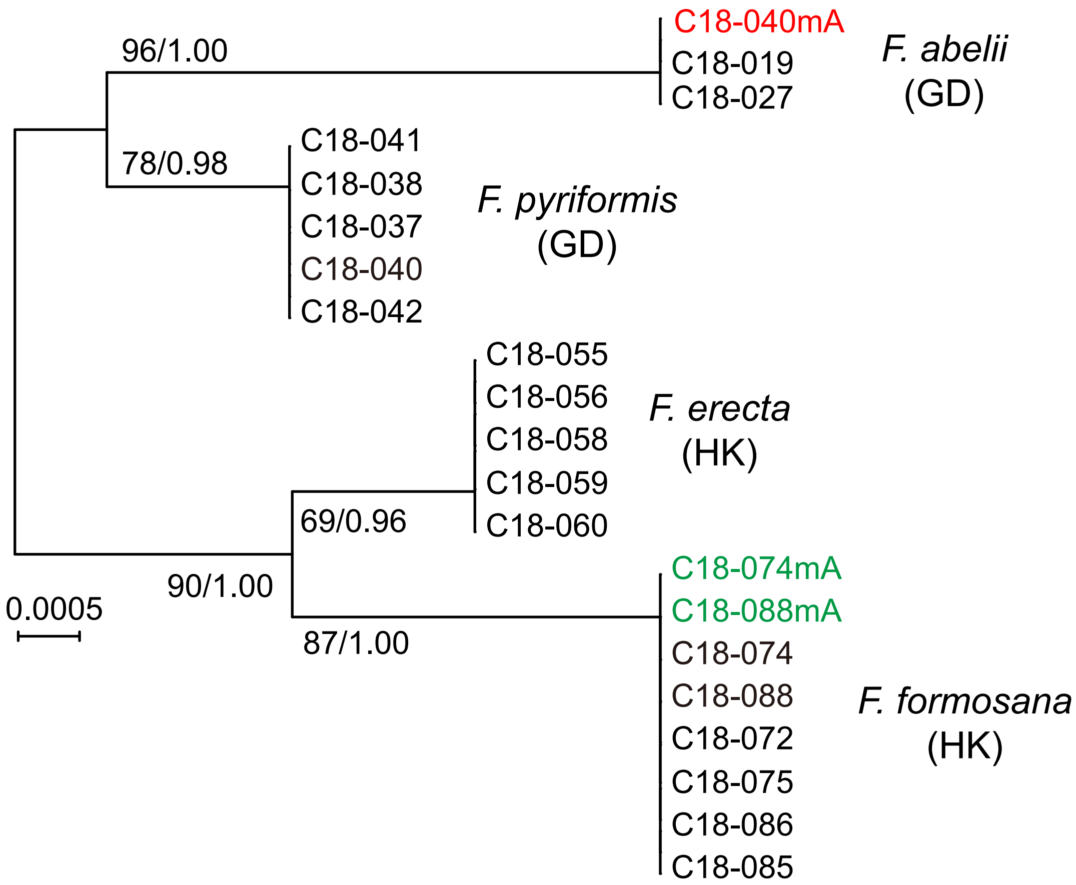

Supplementary Figure 3. ML tree of ITS sequence (671 bp) from leaves and pollen (red and green, respectively) of four fig species. BI analysis produced the same tree topology. The ML bootstrap values and BI posterior probabilities are indicated at the tree nodes (ML/BI). The sample names show the fig individuals from which leaves were collected, but the sample names indicated in red and green show the pollinator foundresses from which pollens was sampled. C18-040mA (red), C18-074mA (green) and C18-088mA (green) were collected from the inside of receptive syconia from *F. pyriformis* (C18-040) and *F. formosana* (C18-074 and C18-088). For sample information, refer to Supplementary Data 1. Species names and population names are shown to the right side of the tree. The results show that the pollinator wasp C18-040mA collected from inside the receptive syconia of *F. pyriformis* carried pollen of *F. abelii*. In other words, pollinator wasps that are reared in *F. abelii* can enter the syconia of *F. pyriformis*.

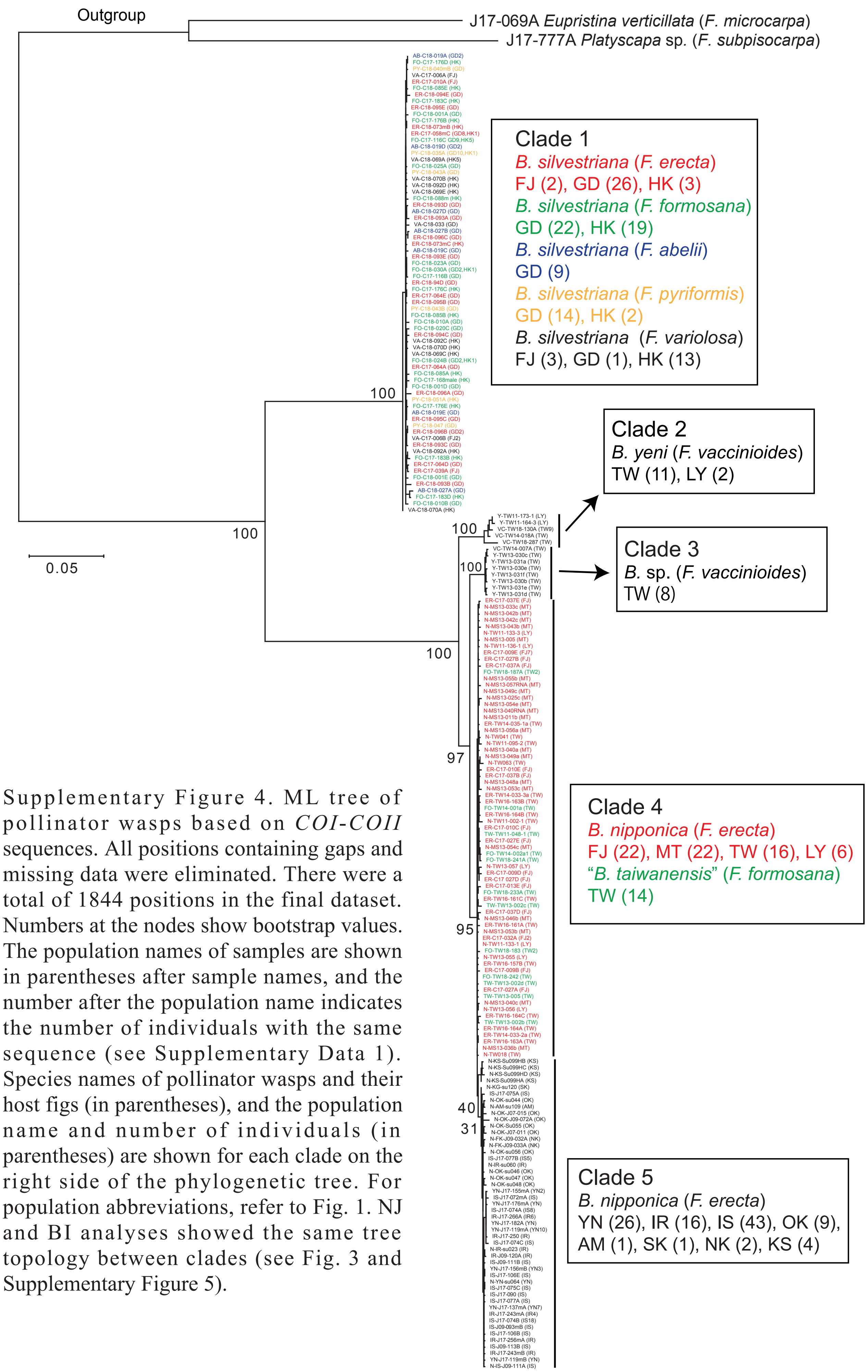

Supplementary Figure 4. ML tree of pollinator wasps based on *COI-COII* sequences. All positions containing gaps and missing data were eliminated. There were a total of 1844 positions in the final dataset. Numbers at the nodes show bootstrap values. The population names of samples are shown in parentheses after sample names, and the number after the population name indicates the number of individuals with the same sequence (see Supplementary Data 1). Species names of pollinator wasps and their host figs (in parentheses), and the population name and number of individuals (in parentheses) are shown for each clade on the right side of the phylogenetic tree. For population abbreviations, refer to Fig. 1. NJ and BI analyses showed the same tree topology between clades (see Fig. 3 and Supplementary Figure 5).

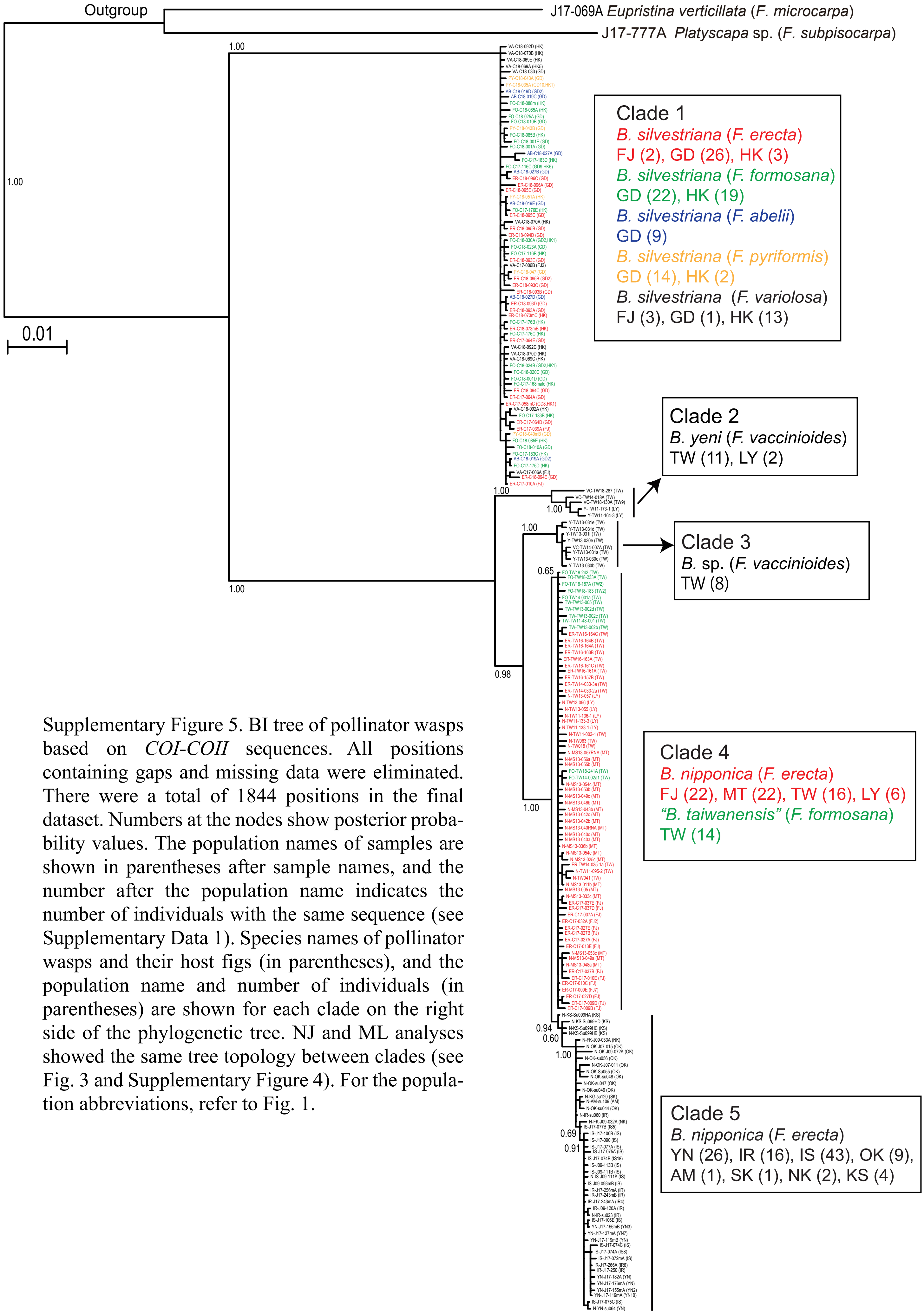

Supplementary Figure 5. BI tree of pollinator wasps based on *COI-COII* sequences. All positions containing gaps and missing data were eliminated. There were a total of 1844 positions in the final dataset. Numbers at the nodes show posterior probability values. The population names of samples are shown in parentheses after sample names, and the number after the population name indicates the number of individuals with the same sequence (see Supplementary Data 1). Species names of pollinator wasps and their host figs (in parentheses), and the population name and number of individuals (in parentheses) are shown for each clade on the right side of the phylogenetic tree. NJ and ML analyses showed the same tree topology between clades (see Fig. 3 and Supplementary Figure 4). For the population abbreviations, refer to Fig. 1.

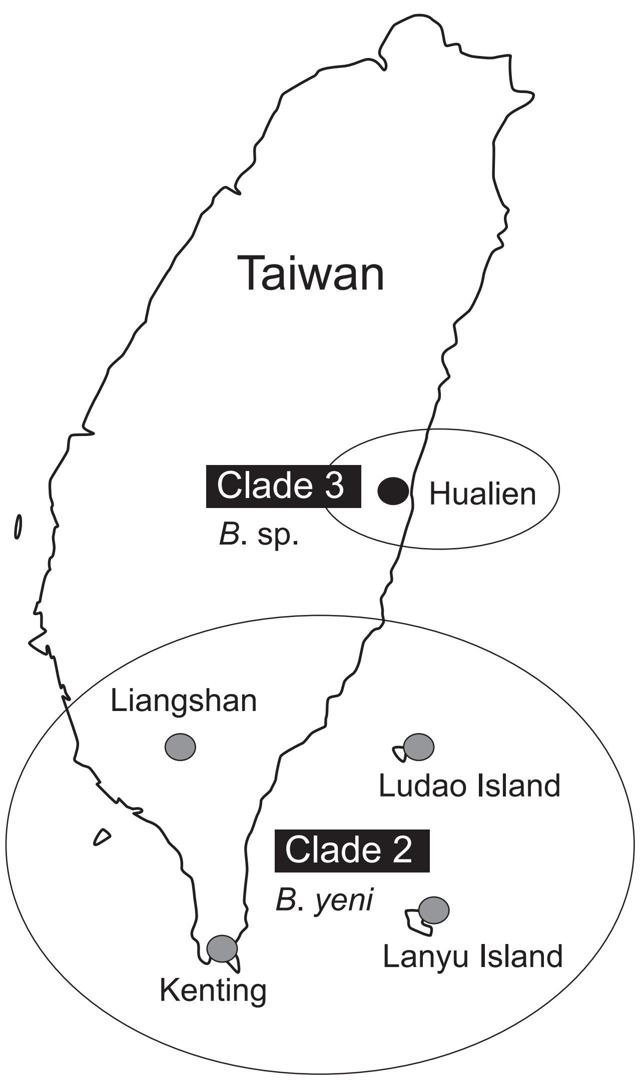

Supplementary Figure 6. Sample localities of the pollinator wasps (*Blastophaga yeni* and *B*. sp.) associated with *F. vaccinioides*. The two genetically distinct clades (Clades 2 and 3 in Fig. 3) are indicated.

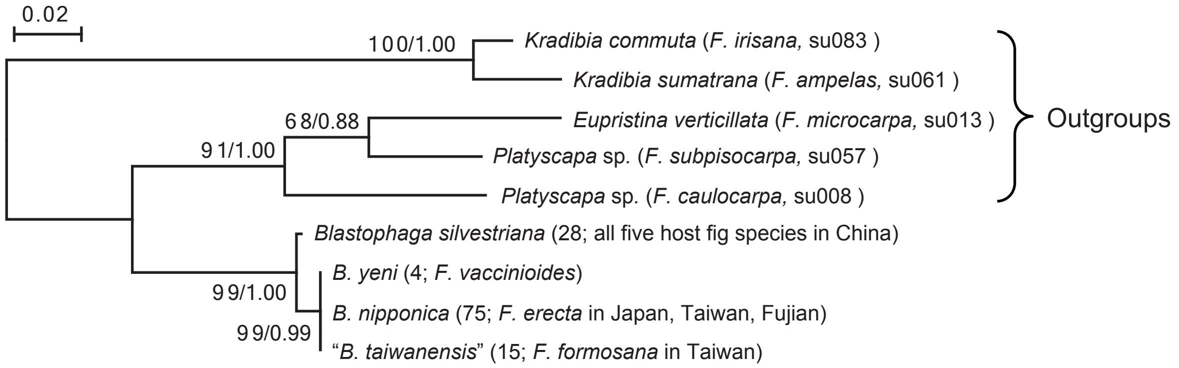

Supplementary Figure 7. ML tree of pollinator wasps inferred by nuclear 28S rDNA sequences. Five species were used as outgroups for the phylogenetic analyses. All positions containing gaps and missing data were eliminated. There was a total of 830 positions in the final dataset. The host fig species are shown in parentheses with the number indicating the wasp individuals with the same sequence. BI analysis produced the same tree topology. ML bootstrap values/BI posterior probabilities are indicated at tree nodes.

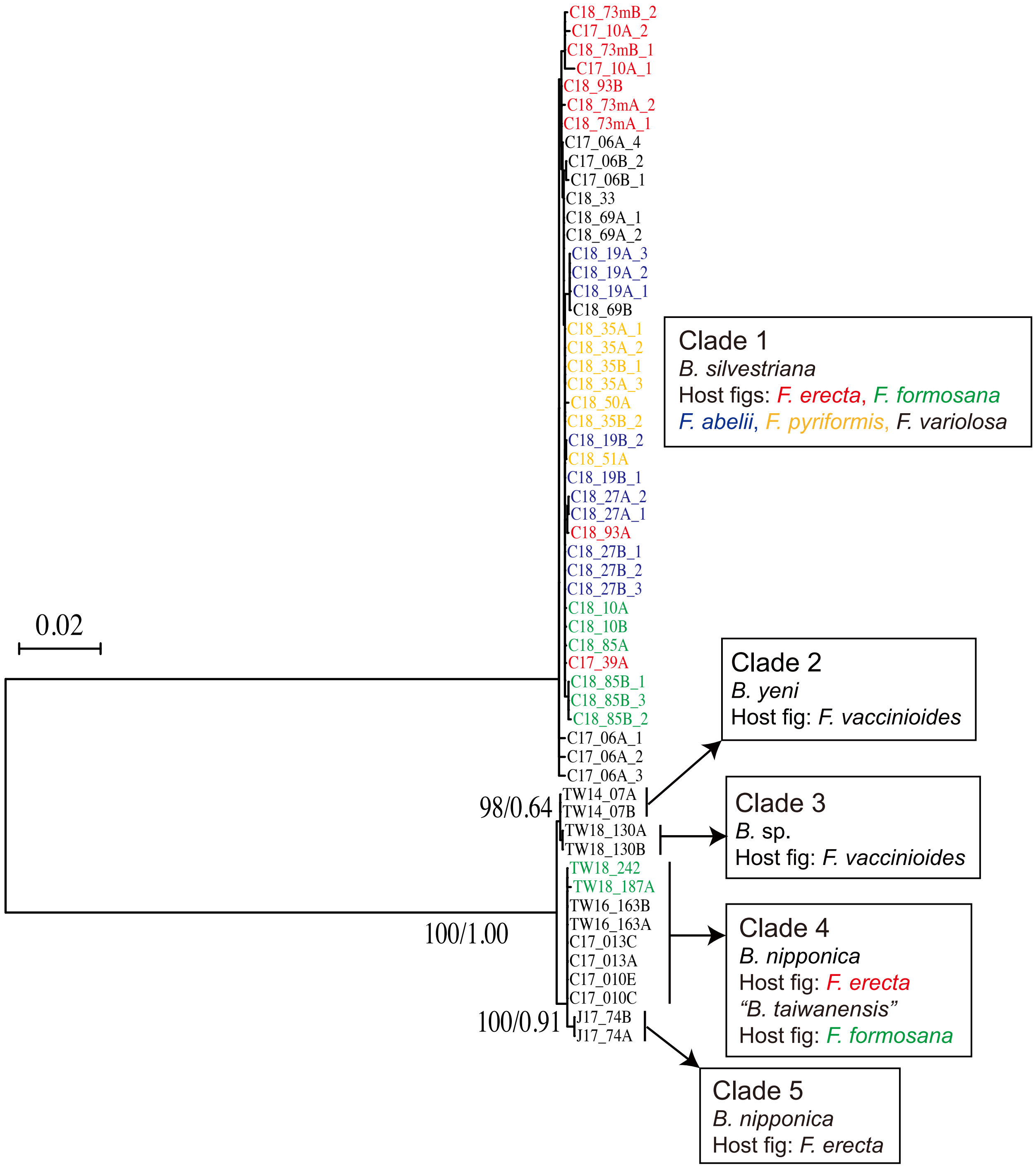

Supplementary Figure 8. ML tree of pollinator wasps based on ITS1 sequences. There were a total of 1712 positions in the final dataset. BI analysis gave the same topology, except for some small differences within clades (tree not shown). ML bootstrap values/BI posterior probabilities are indicated at tree nodes. The phylogenetic clades correspond to that shown in Fig. 3.

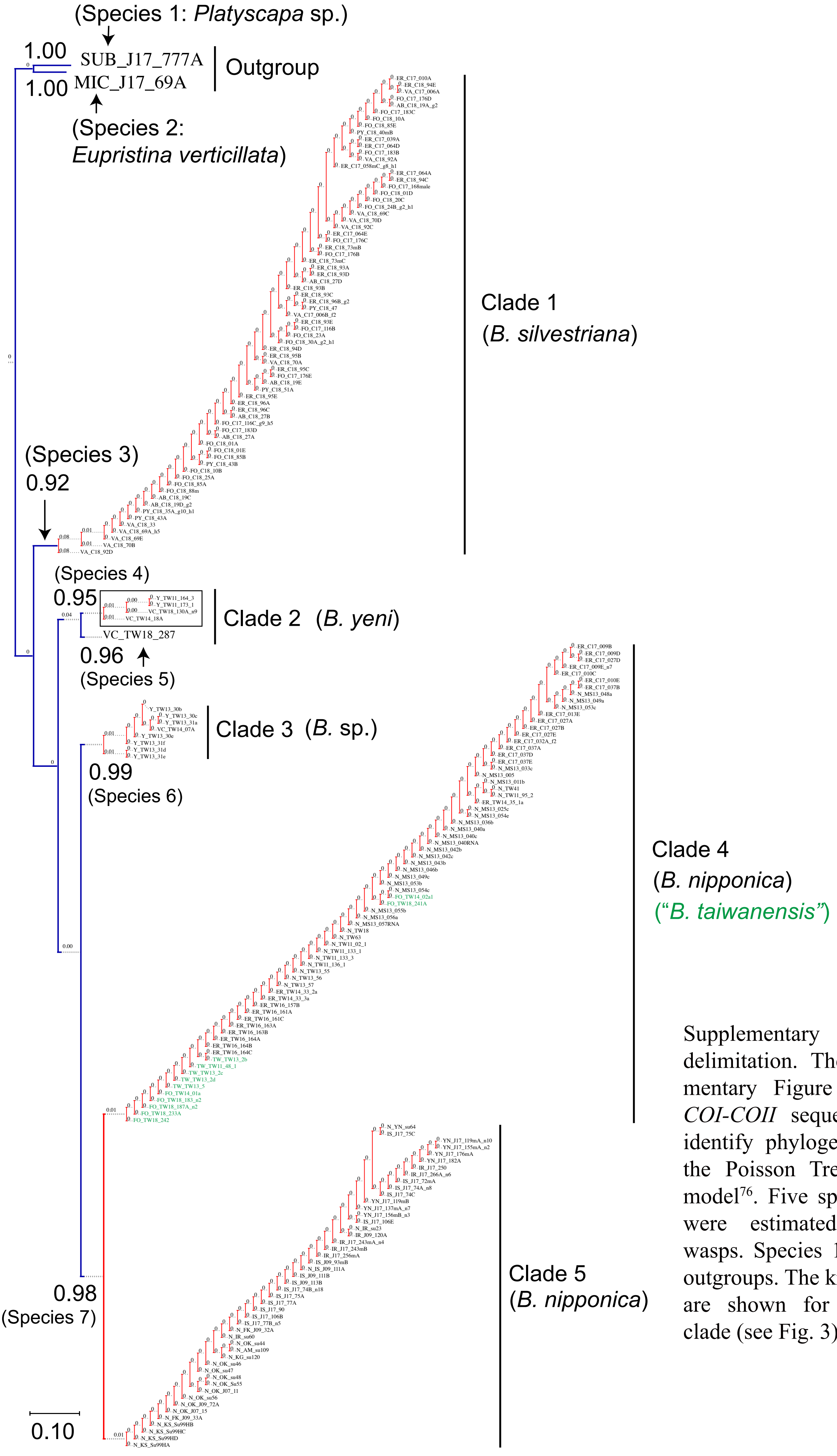

Supplementary Figure 9. Species delimitation. The BI tree (Supplementary Figure 5) inferred using *COI-COII* sequences was used to identify phylogenetic species using the Poisson Tree Processes (PTP) model[76]. Five species (Species 3~7) were estimated for Blastophaga wasps. Species 1 and Species 2 are outgroups. The known species names are shown for each phylogenetic clade (see Fig. 3).

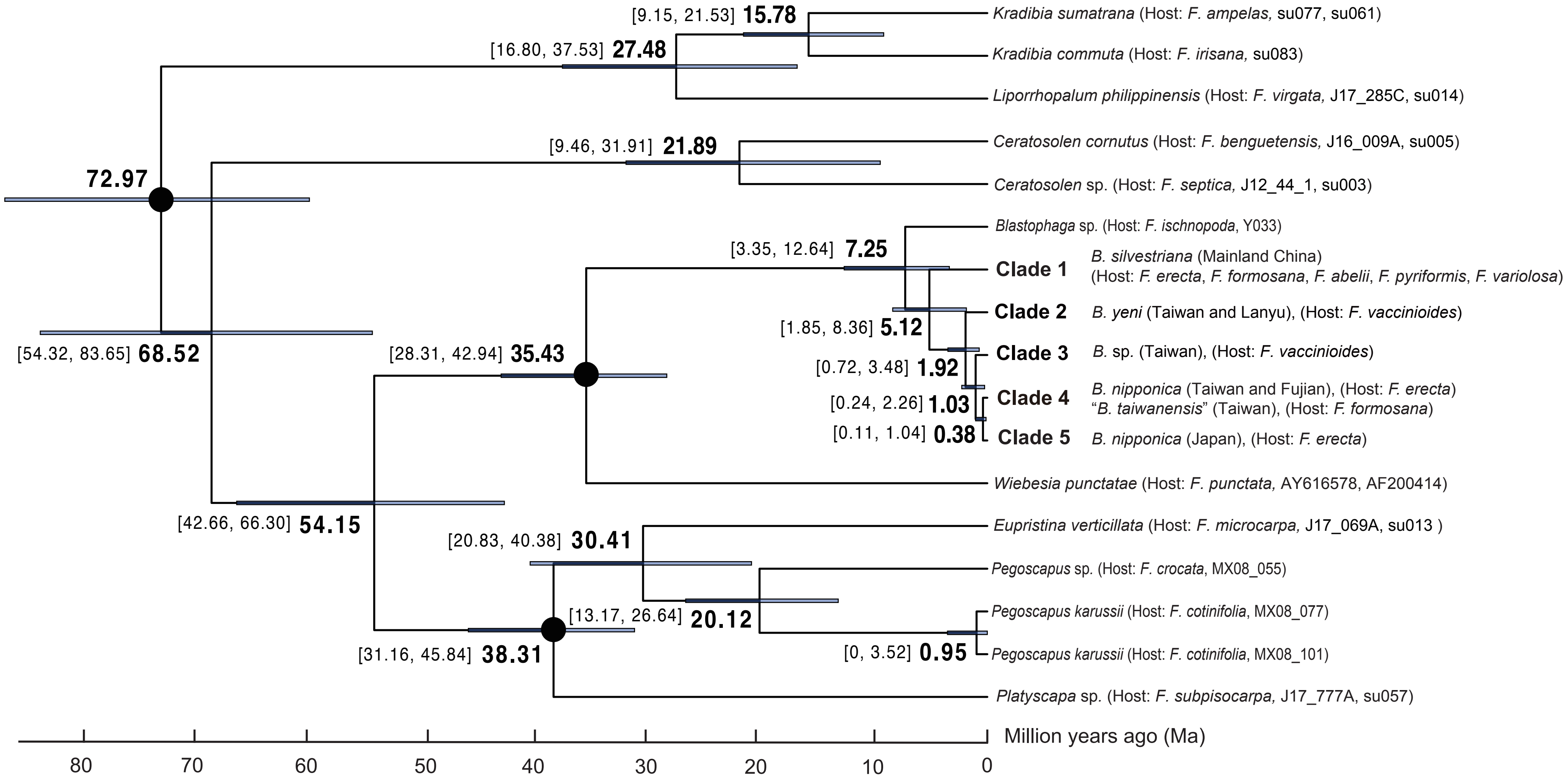

Supplementary Figure 10. The topology and chronogram based on 28S rDNA and *COI-COII* sequences of pollinator wasps. The analysis was performed with BEAST. Grey bars around nodes indicate the 95% highest posterior density (HPD) intervals. The divergence time (mean) and CI (confidence intervals) were shown at the nodes. Black circles at three nodes show calibration points (refer to Methods).

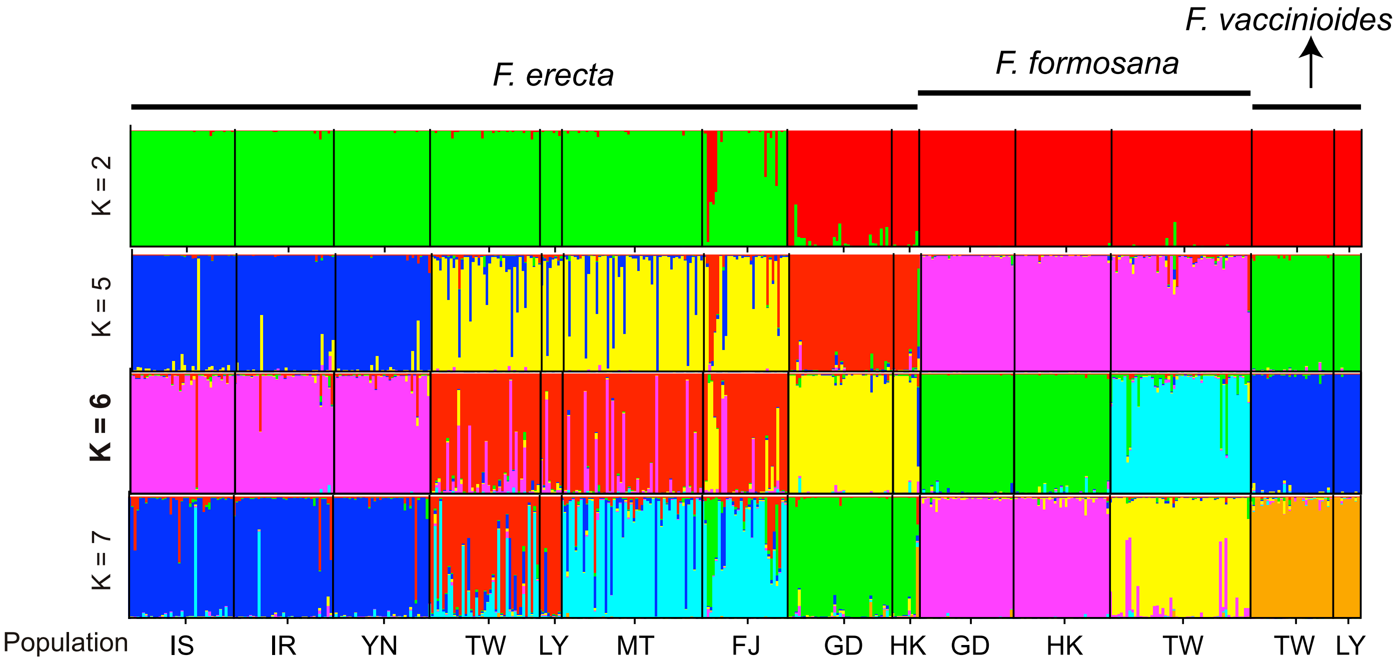

Supplementary Figure 11. Population genetic structure of figs based on SSR data. SSR data were genotyped from nine loci. *K*=6 was the most likely *K*. Structures with *K*=2, *K*=5, and *K*=7 are also shown (refer to results). The samples used in these analyses are shown in Table 1 and Supplementary Data 1. For the abbreviations of population names, refer to Fig. 1.

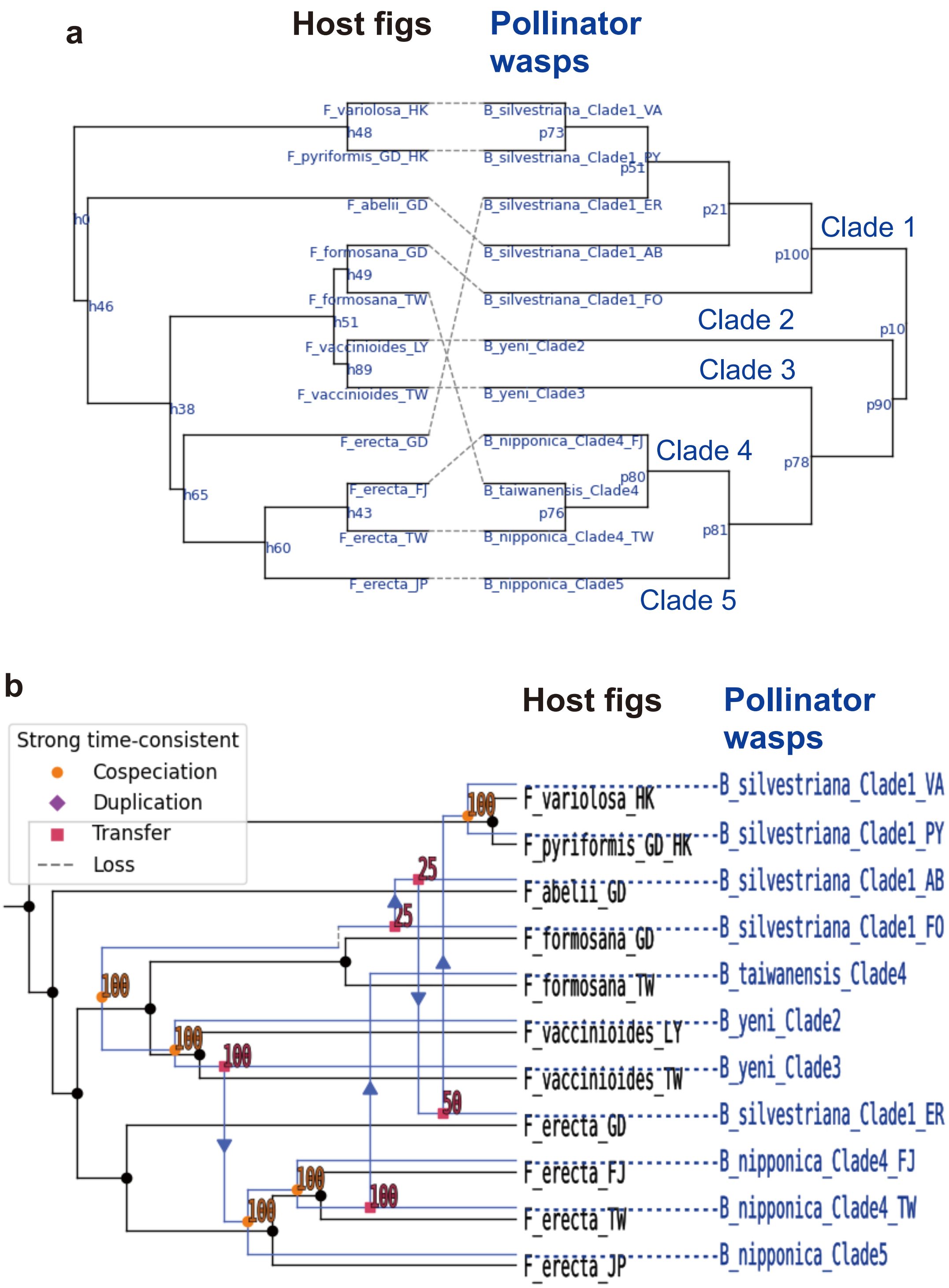

Supplementary Figure 12. Cophylogenetic analysis between figs and pollinator wasps. a, The input phylogenetic trees of figs and pollinators used for cophylogenetic analysis. The tree topologies are based on the results of phylogenetic analyses (Figs. 2 and 3) and population genetic analyses (Fig. 4a and 4d, and Supplementary Tables 1 and 2). These tree topologies are the same to those shown in Fig. 5, except for the *B. silvestriana* wasps within Clade 1. Although these wasps are genetically indistinguishable (Fig. 3), each host-pollinator association between *B. silvestriana* and its five host fig species was used in the analysis because the software does not allow multiple host species to be associated with one pollinator species. b, Results of cophylogenetic analysis by the eMPRess GUI Version 1.0[75]. The evolutionary events (Cospeciation, Duplication, Transfer, and Loss) and supporting values are shown. The two upper-case letters after fig species names indicate the abbreviation of the pupulation name (see Fig. 1). The two uppercase letters after the wasp species name (B_silvestriana) indicate the host species: VA, *F. variolosa*; PY, *F. pyriformis*; AB, *F. abelii*; FO, *F. formosana*; ER, *F. erecta*.

Supplementary Table 1. Pairwise F_{ST} values between fig populations analyzed based on SSR data using Arlquin v3.5[68].

	e-IS	e-IR	e-YN	e-TW	e-MT	e-FJ	e-GD	f-GD	f-HK	f-TW	v-TW
e-IS (n=38)		0.036	*	*	*	*	*	*	*	*	*
e-IR (n=36)	0.008		*	*	*	*	*	*	*	*	*
e-YN (n=35)	0.025	0.019		*	*	*	*	*	*	*	*
e-TW (n=40)	0.081	0.077	0.083		*	*	*	*	*	*	*
e-MT (n=51)	0.093	0.108	0.099	0.056		*	*	*	*	*	*
e-FJ (n=31)	0.081	0.092	0.102	0.05	0.025		*	*	*	*	*
e-GD (n=38)	0.18	0.196	0.208	0.146	0.165	0.126		*	*	*	*
f-GD (n=34)	0.297	0.333	0.325	0.334	0.321	0.316	0.312		0.018	*	*
f-HK (n=35)	0.3	0.334	0.329	0.34	0.333	0.326	0.314	0.019		*	*
f-TW (n=51)	0.256	0.285	0.272	0.279	0.27	0.259	0.245	0.123	0.136		*
v-TW (n=30)	0.267	0.287	0.28	0.262	0.269	0.258	0.243	0.267	0.276	0.2	

p-value: * < 0.001

e, *Ficus erecta*; f, *F. formosana*; v, *F. vaccinioides*

Population abbreviations: IS, Ishigaki Island; IR, Iriomote Island; YN, Yonaguni Island; TW, Taiwan; MT, Matsu Islands; FJ, Fujian; GD, Guangdong; HK, Hong Kong.

Supplementary Table 2. Pairwise F_{ST} values between pollinator wasp populations analyzed based on SSR data using Arlquin v3.5[68].

	n-IS	n-YN	n-TW	n-MT	n-FJ	t-TW	y-TW
n-IS (n=42)		0.369	*	*	*	*	*
n-YN (n=25)	0.008		*	*	*	*	*
n-TW (n=36)	0.217	0.22		*	*	*	*
n-MT (n=47)	0.31	0.309	0.113		0.198	*	*
n-FJ (n=22)	0.323	0.326	0.111	0.01		*	*
t-TW (n=30)	0.231	0.228	0.021	0.069	0.075		*
y-TW (n=34)	0.29	0.278	0.273	0.343	0.347	0.273	

p-value: * < 0.001

n, *Blastophaga nipponica*; t, "*B. taiwanensis*"; y, *B. yeni/B*. sp.

For population abbreviations, refer to Fig. 1 and Supplementary Table 1.

Supplementary Table 3. Primers used for PCR amplification in this study.

Primer name	Sequence	Direction	Reference
For 28S rDNA			
28S-01	GACTACCCCCTGAATTTAAGCAT	Forward	Su et al.[53]
28SR-01	GACTCCTTGGTCCGTGTTTCAAG	Reverse	"
This primer pair amplified the 28S rDNA region from all fig wasp samples.			
For ITS1 of wasps			
18S_F1	GTCGCTACTACCGATTGAATGA	Forward	This study
5.8S_R1	GTGCGTTCGAAATGTCGATGTTCA	Reverse	This study
This primer pair was used for *B. nipponica*, "*B. taiwanensis*", *B. yeni*, and *B. silvestriana* (Ref. Supplementary Fig. S8).			
For COI-COII			
COI1-1-ple	TTAATTGGAAATGATCAAATTTATAAT	Forward	Su et al.[32]
COII-BNR	ATCGAATTTGACAATTTATTGG	Reverse	This study
COI-2M	ACATAATGAAAATGTGCTACTACATAATA	Reverse	Su et al.[32]
COI-2MbengF	TATTATGTTGTTGCTCATTTTCATTATGT	Forward	This study

COI1-1-ple / BNR pair was used for *B. nipponica*, *"B. taiwanensis"*, and *B. yeni*. COI1-1-ple / COI-2M and COI-2MbengF / COII-BNR pairs were used for *B. silvestriana*.			
For plastid DNA			
C1F	CCC CCT AGA AAC GTA TAG GA	Forward	Azuma et al.[16]
C1R	ATA GTC CAT GAT GGA GCT CG	Reverse	"
C2F	GGT AAA AGT GTG ATT CGT TC	Forward	"
C2R	GTT TCA TTC GGC TCC TTT AT	Reverse	"
C3F	AGA GAT GGT TCT ACT TCG TC	Forward	"
C3R	ACT TTC ATC TCG TAC AGC TC	Reverse	"
C5F	CGA AAT CGG TAG ACG CTA CG	Forward	"
C5R	GGG GAT AGA GGG ACT TGA AC	Reverse	"
C6F	GGT TCA AGT CCC TCT ATC CC	Forward	"
C6R	ATT TGA ACT GGT GAC ACG AG	Reverse	"
C9F	AGA ACC AGA AGT AGT AGG AT	Forward	"
C9R	ACA CCA GCT TTG AAT CCA AC	Reverse	"
C1F-C1R for *rps16* intron; C2F-C2R for *trnG* intron; C3F-C3R for *petB* intron; C5F-C5R for *trnL* intron; C6F-C6R for *trnL-trnF* intergenic spacer; C9F-C9R for *atpB-rbcL* intergenic spacer.			
For ITS of figs			
ITS-Y5	TAGAGGAAGGAGAAGTCGTAACAA	Forward	Azuma et al.[16]

ITS-Y4	CCCGCCTGACCTGGGGTCGC	Reverse	"
This primer pair was used for amplification of ITS region from fig leaves and pollens (Ref. Supplementary Fig. S3).			
For SSR of figs			
Frac241	GGCTCAAGCAAGGGATGGA	Forward	Crozier et al.[62]
	CGAGCTCATTTGTTTATCGACC	Reverse	
Frub061	GTACACTCTCTTAGCTGCC	Forward	"
	TACCTTTCTCTGGACATTC	Reverse	
Frub391	AGATGTCAAATAAGGTCAGCT	Forward	"
	AGATGCAGTTCCATACAATTC	Reverse	
Frub416	CAGCAATGATCTTGACCT	Forward	"
	GTACTCATCAATATCTCTAAACAAC	Reverse	
Frac222	ACCCATATAGCAGTCTTCAGA	Forward	"
	GCTTGTTGACTCCGCAACTA	Reverse	
FinsM5	ATGAATGGTGAAATCCTGAA	Forward	Vignes et al.[63]
	CATGGCCTCAACTTAGAAAC	Reverse	
FinsQ5	CATGTCAGGAGGTGTCTAGG	Forward	"
	CCAAATGGGTATGTCAAG	Reverse	
FM1-27	GTGATTTGCGATGGCGTGGTTTA	Forward	Zavodna et al.[64]

	CGCTTGCTCGTCAGTGTCC	Reverse	
	CGATCTCGACGACGACTACA	Forward	
FereACOI	GTGTGGAATTTTCGATAGGC	Reverse	This study
For SSR of pollinator wasps			
	GACATACCATCTCCTCTTCC	Forward	
Ega01	GCGGAAAAATCCCACAACTC	Reverse	Kusumi & Su[74]
	TAGCTCCTGAGAGTGAGAGC	Forward	
Ega03	TTCACATCCATTACTTGAAAAG	Reverse	"
	ATTTATCAGCGTAGCGAGAG	Forward	
Ega06	GCGTAACGGTAAGGCTGTTC	Reverse	"
	CTAAGCTCCCAGTAATTTTCAC	Forward	
Ega07	AGTGCAGCAGTCAACGACAC	Reverse	"
	CCTCTTAGAAAGCATGACTC	Forward	
Ega08	TGATACATGGACATCGTG	Reverse	"
	TAATACAAAAGAGACAGTAGAAGG	Forward	
Ega09	ACTCCTTATCCTTGCCGTTTC	Reverse	"
	CAGTGAGGTACAACGAATAGAG	Forward	
Ega10	GGCTTTTCTTGGAACGACAG	Reverse	"
Ega11	AAATTACTCGACGCCTACATACC	Forward	"

	TCTTGTTCCTTTTCGATGTGA	Reverse	
Hga04	GGAGTGAGTACCTTGGTTAGAG	Forward	"
	GGATCGTTACCAGCACCAAT	Reverse	
Hga09	AAGTGTGAAGGGATTAGAGTGA	Forward	"
	GGTTTCATAGGCATTTTTCTT	Reverse	
Hga10	CAGATTCAGAAAAAACGGCG	Forward	"
	ATGAGCGACTGAGAGAGTGT	Reverse	
Eca15	TTCAGCGACGGTAAGTTCTG	Forward	"
	ACTCTTCGAGGGGGTTAAAT	Reverse	
Eca22	TGCATATGTGTATAATGAGCA	Forward	"
	ATAACGTACTCCTGGAAAATTA	Reverse	
Hca07	ATATGCACTTTTATTGGAACC	Forward	"
	GGGCCACACAATAATCCAAC	Reverse	
Hca19	GAGGCATTTAATGAATCCTCT	Forward	"
	AAACGATACTTGCAACGAAG	Reverse	

Supplementary Table 4. PCR amplification conditions.

For plastide DNA (*rps16* intron, *trnG* intron, *trnL* intron, and *trnL-trnF* intergenic spacer) of figs, 28S rDNA and ITS1 of pollinator wasps.		
94 ℃	1 min	
95 ℃	20 sec	35 cycles
58 ℃	10 sec	
68 ℃	2 min	
72 ℃	10 min	
4 ℃	Stored	
For plastide DNA (*petB* intron and *atpB-rbcL* intergenic spacer) of figs.		
94 ℃	5 min	
94 ℃	1 min	35 cycles
50 ℃	1 min 30 sec	
72 ℃	2 min	
72 ℃	7 min	
4 ℃	Stored	
For ITS of figs.		
94 ℃	5 min	
94 ℃	30 sec	35 cycles
50 ℃	30 sec	

72 ℃	1 min				
72 ℃	7 min				
4 ℃	Stored				
For mtDNA (COI-COII) of pollinator wasps.					
COI1-1-ple / COI-2M and COI-2MbengF / COII-BNR			COI1-1-ple / COII-BNR		
94 ℃	5 min		94 ℃	5 min	
94 ℃	30 sec	50 cycles	94 ℃	30 sec	40 cycles
50 ℃	1 min		48 ℃	1 min	
70 ℃	2 min		70 ℃	1 min	
70 ℃	7 min		70 ℃	10 min	
4 ℃	Stored		4 ℃	Stored	
For SSR.					
Figs: fluorescent primer			Fig wasps: with M13 primer as an adaptor		
94 ℃	15 min		95 ℃	5 min	
94 ℃	30 sec	35-40 cycles	95 ℃	30 sec	20 cycles
57 ℃	90 sec		58 ℃	1 min 30 sec	
72 ℃	1 min		72 ℃	1 min	
72 ℃	10 min		95 ℃	30 sec	30 cycles
4 ℃	Stored		55 ℃	1 min	

	72 ℃	1 min
	72 ℃	5 min
	4 ℃	Stored
