# Supplementary material for: Pollinator sharing, copollination, and speciation by host shifting among six closely related dioecious fig species
Source: Commun Biol. 2022 Apr 8;5:284. doi: 10.1038/s42003-022-03223-0 (PMC8993897; doi:10.1038/s42003-022-03223-0)
Supplement: Supplementary file 3 — Description of Additional Supplementary Files [file 42003_2022_3223_MOESM3_ESM.pdf]

Description of Additional Supplementary Files

File name: Supplementary Data 1
Description: Detailed sample list and data information used in this study.
