# Supplementary material for: Pollinator sharing, copollination, and speciation by host shifting among six closely related dioecious fig species
Source: Commun Biol. 2022 Apr 8;5:284. doi: 10.1038/s42003-022-03223-0 (PMC8993897; doi:10.1038/s42003-022-03223-0)
Supplement: Supplementary file 4 — Supplementary Data 1 [file 42003_2022_3223_MOESM4_ESM.docx]

| Supplementary Data 1. Detailed sample list and data information used in this study. | | | | | | | | | | | | | | |
| --- | --- | --- | --- | --- | --- | --- | --- | --- | --- | --- | --- | --- | --- | --- |
| Host figs | | Pollinator wasps | | Population | Locality | Fig data | | | | Pollinator data | | | | Sampling date |
| Species | Sample ID | Sample ID | Species |  |  | Plastid | ITS | Mig-seq | SSR | 28S | ITS1 | COI-COII | SSR |  |
|  |  |  |  |  |  |  |  |  |  |  |  |  |  |  |
| *F. erecta* | su099 | su099HA | *B. nipponica* | KS | Botanical Garden, Kyoto University, Kyoto, Japan | h1 |  |  |  | ● |  | ● |  | 2002.07.20 |
|  |  | su099HB | *B. nipponica* | KS |  |  |  |  |  | ● |  | ● |  | 2002.07.20 |
|  |  | su099HC | *B. nipponica* | KS |  |  |  |  |  | ● |  | ● |  | 2002.07.20 |
|  |  | su099HD | *B. nipponica* | KS |  |  |  |  |  | ● |  | ● |  | 2002.07.20 |
|  | J09-32 | J09-032A | *B. nipponica* | NK | Futamigaura, Itoshima, Fukuoka, Japan | h1 |  |  |  | ● |  | ● |  | 2009.05.29 |
|  | J09-33 | J09-033A | *B. nipponica* | NK | Futamigaura, Itoshima, Fukuoka, Japan | h1 |  |  |  | ● |  | ● |  | 2009.05.29 |
|  | su120 | su120 | *B. nipponica* | SK | Kamiyaku-cho, Kagoshima, Japan | h1 |  |  |  | ● |  | ● |  | 2002.09.09 |
|  | su109 | su109 | *B. nipponica* | AM | Asato, Naze, Kagoshima, Japan | h1 |  |  |  | ● |  | ● |  | 2002.09.12 |
|  | su044 | su044 | *B. nipponica* | OK | Tengu-iwa, Kitadaito, Okinaw, Japan | h1 |  |  |  | ● |  | ● |  | 2002.05.21 |
|  | su046 | su046 | *B. nipponica* | OK | Kenmin-no-mori, Onnna-son, Okinawa, Japan | h2 |  |  |  | ● |  | ● |  | 2002.05.24 |
|  | su047 | su047 | *B. nipponica* | OK | Kenmin-no-mori, Onnna-son, Okinawa, Japan | h1 |  |  |  | ● |  | ● |  | 2002.05.24 |
|  | su048 | su048 | *B. nipponica* | OK | Mt. Nago-dake, Nago, Okinawa, Japan | h1 |  |  |  | ● |  | ● |  | 2002.05.24 |
|  | su055 | su055 | *B. nipponica* | OK | Nanjyo, Okinawa, Japan | h1 |  |  |  | ● |  | ● |  | 2002.05.26 |
|  | su056 | su056 | *B. nipponica* | OK | Nanjyo, Okinawa, Japan | h1 |  |  |  | ● |  | ● |  | 2002.05.26 |
|  | J07-011 | J07-011 (suJ11) | *B. nipponica* | OK | Okoku, Nanjyo, Okinawa, Japan | h1 |  |  |  | ● |  | ● |  | 2007.02.24 |
|  | J07-015 | J07-015 (suJ15) | *B. nipponica* | OK | Tina, Nanjyo, Okinawa, Japan | h1 |  |  |  | ● |  | ● |  | 2007.02.24 |
|  | J09-072 | J09-072A | *B. nipponica* | OK | Tinen, Nanjyo, Okinawa, Japan | h1 |  |  |  | ● |  | ● |  | 2009.06.08 |
| Sub-total | 14 | 17 |  |  |  | 14 |  |  |  | 17 |  | 17 |  |  |
|  | Ishi76 | su076 |  | IS | Inoda, Ishigaki, Okinawa , Japan | h3 |  |  | ● | ● |  |  |  | 2002.06.03 |
|  | Ishi87 | su087 |  | IS | Mt. Omoto-dake, Ishigaki, Okinawa, Japan | h4 |  |  | ● | ● |  | ● |  | 2002.06.05 |
|  | Ishi90 | su090 |  | IS | Mt. Omoto-dake, Ishigaki, Okinawa, Japan | h4 |  |  | ● | ● |  | ● |  | 2002.06.05 |
|  | J09-092 | J09-092A | *B. nipponica* | IS | Banna Park, Ishigaki, Okinawa, Japan |  |  |  | ● |  |  | ○ | ● | 2009.10.02 |
|  |  | J09-092B | *B. nipponica* | IS |  |  |  |  |  |  |  | ○ | ● |  |
|  | J09-093 | J09-093mA | *B. nipponica* | IS | Banna Park, Ishigaki, Okinawa, Japan |  |  |  |  |  |  |  | ● |  |
|  |  | J09-093mB | *B. nipponica* | IS |  |  |  |  |  |  |  | ○ | ● |  |
|  | J09-094 | J09-094mA | *B. nipponica* | IS | Banna Park, Ishigaki, Okinawa, Japan |  |  |  | ● |  |  | ○ | ● | 2009.10.02 |
|  |  | J09-094mB | *B. nipponica* | IS |  |  |  |  |  |  |  | ○ | ● |  |
|  | J09-095 |  |  | IS | Banna Park, Ishigaki, Okinawa, Japan |  |  |  | ● |  |  |  |  | 2009.10.02 |
|  | J09-096 | J09-096A | *B. nipponica* | IS | Banna Park, Ishigaki, Okinawa, Japan |  |  |  | ● |  |  | ○ | ● | 2009.10.02 |
|  | J09-097 |  |  | IS | Sakieda, Ishigaki, Okinawa, Japan |  |  |  | ● |  |  |  |  | 2009.10.02 |
|  | J09-098 |  |  | IS | Sakieda, Ishigaki, Okinawa, Japan |  |  |  | ● |  |  |  |  | 2009.10.02 |
|  | J09-099 |  |  | IS | Sakieda, Ishigaki, Okinawa, Japan |  |  |  | ● |  |  |  |  | 2009.10.02 |
|  | J09-100 |  |  | IS | Kabira, Ishigaki, Okinawa, Japan |  |  |  | ● |  |  |  |  | 2009.10.02 |
|  | J09-101 | J09-101A | *B. nipponica* | IS | Yonehara Beach, Ishigaki, Okinawa, Japan |  |  |  | ● |  |  | ○ | ● | 2009.10.02 |
|  |  | J09-101B | *B. nipponica* | IS |  |  |  |  |  |  |  | ○ | ● |  |
|  |  | J09-101C | *B. nipponica* | IS |  |  |  |  |  |  |  | ○ | ● |  |
|  | J09-102 | J09-102A | *B. nipponica* | IS | Yonehara Beach, Ishigaki, Okinawa, Japan |  |  |  | ● |  |  | ○ | ● | 2009.10.02 |
|  | J09-103 |  |  | IS | Oozato, Ishigaki, Okinawa, Japan |  |  |  | ● |  |  |  |  | 2009.10.03 |
|  | J09-104 |  |  | IS | Oozato, Ishigaki, Okinawa, Japan |  |  |  | ● |  |  |  |  | 2009.10.03 |
|  | J09-105 |  |  | IS | Nobaru, Ishigaki, Okinawa, Japan |  |  |  | ● |  |  |  |  | 2009.10.03 |
|  | J09-106 |  |  | IS | Nobaru, Ishigaki, Okinawa, Japan |  |  |  | ● |  |  |  |  | 2009.10.03 |
|  | J09-107 |  |  | IS | Hirakubosaki lighthouse, Ishigaki, Okinawa, Japan |  |  |  | ● |  |  |  |  | 2009.10.03 |
|  | J09-109 |  |  | IS | Hirakubosaki lighthouse, Ishigaki, Okinawa, Japan |  |  |  | ● |  |  |  |  | 2009.10.03 |
|  | J09-110 |  |  | IS | Nosoko, Ishigaki, Okinawa, Japan |  |  |  | ● |  |  |  |  | 2009.10.03 |
|  | J09-111 | J09-111A | *B. nipponica* | IS | Ishigaki Dam, Ishigaki, Okinawa, Japan |  |  |  | ● |  |  | ● | ● | 2009.10.03 |
|  |  | J09-111B | *B. nipponica* | IS |  |  |  |  |  |  |  | ○ | ● |  |
|  | J09-112 |  |  | IS | Ishigaki Dam, Ishigaki, Okinawa, Japan |  |  |  | ● |  |  |  |  | 2009.10.03 |
|  | J09-113 | J09-113A | *B. nipponica* | IS | Ishigakijima Astronomical Observatory, Ishigaki, Okinawa, Japan |  |  |  | ● |  |  | ○ | ● | 2009.10.03 |
|  |  | J09-113B | *B. nipponica* | IS |  |  |  |  |  |  |  | ○ | ● |  |
|  | J09-114 | J09-114mA | *B. nipponica* | IS | Ishigakijima Astronomical Observatory, Ishigaki, Okinawa, Japan |  |  |  | ● |  |  | ○ | ● | 2009.10.03 |
|  | J09-115 | J09-115A | *B. nipponica* | IS | Sukuji Beach, Ishigaki, Okinawa, Japan |  |  |  | ● |  |  | ○ | ● | 2009.10.04 |
|  | J09-116 |  |  | IS | Kabira, Ishigaki, Okinawa, Japan |  |  |  | ● |  |  |  |  | 2009.10.04 |
|  | J09-117 |  |  | IS | Sokobaru Dam, Ishigaki, Okinawa, Japan |  |  |  | ● |  |  |  |  | 2009.10.04 |
|  | J17-072 | J17-072mA | *B. nipponica* | IS | Hirakubosaki lighthouse, Ishigaki, Okinawa, Japan | h4 |  |  | ○ |  |  | ○ | ○ | 2017.04.22 |
|  | J17-074 | J17-074A | *B. nipponica* | IS | Hirakubosaki lighthouse, Ishigaki, Okinawa, Japan | h4 |  |  | ○ |  | ○ | ○ | ○ | 2017.04.22 |
|  |  | J17-074B | *B. nipponica* | IS |  |  |  |  |  |  | ○ | ○ | ○ |  |
|  |  | J17-074C | *B. nipponica* | IS |  |  |  |  |  |  |  | ○ | ○ |  |
|  | J17-075 | J17-075A | *B. nipponica* | IS | Hirakubosaki lighthouse, Ishigaki, Okinawa, Japan | h4 |  |  | ○ |  |  | ○ | ○ | 2017.04.22 |
|  |  | J17-075B | *B. nipponica* | IS |  |  |  |  |  |  |  | ○ | ○ |  |
|  |  | J17-075C | *B. nipponica* | IS |  |  |  |  |  |  |  | ○ | ○ |  |
|  | J17-077 | J17-077A | *B. nipponica* | IS | Hirakubosaki lighthouse, Ishigaki, Okinawa, Japan | h4 |  |  | ○ |  |  | ○ | ○ | 2017.04.22 |
|  |  | J17-077B | *B. nipponica* | IS |  |  |  |  |  |  |  | ○ | ○ |  |
|  |  | J17-077C | *B. nipponica* | IS |  |  |  |  |  |  |  | ○ | ○ |  |
|  |  | J17-077D | *B. nipponica* | IS |  |  |  |  |  |  |  | ○ | ○ |  |
|  | J17-078 | J17-078A | *B. nipponica* | IS | Hirakubosaki lighthouse, Ishigaki, Okinawa, Japan | h4 |  |  | ○ |  |  | ○ | ○ | 2017.04.22 |
|  |  | J17-078B | *B. nipponica* | IS |  |  |  |  |  |  |  | ○ | ○ |  |
|  |  | J17-078C | *B. nipponica* | IS |  |  |  |  |  |  |  | ○ | ○ |  |
|  |  | J17-078D | *B. nipponica* | IS |  |  |  |  |  |  |  | ○ | ○ |  |
|  |  | J17-078E | *B. nipponica* | IS |  |  |  |  |  |  |  | ○ | ○ |  |
|  | J17-079 | J17-079 | *B. nipponica* | IS | Hirakubosaki lighthouse, Ishigaki, Okinawa, Japan | h4 |  |  | ○ |  |  | ○ | ○ | 2017.04.22 |
|  | J17-090 | J17-090 | *B. nipponica* | IS | Mt. Maese-dake, Ishigaki, Okinawa, Japan | h4 |  | ○ | ○ |  |  | ○ | ○ | 2017.04.23 |
|  | J17-106 | J17-106A | *B. nipponica* | IS | Arakawa, Ishigaki, Okinawa, Japan | h4 |  | ○ | ○ |  |  | ○ | ○ | 2017.04.23 |
|  |  | J17-106B | *B. nipponica* | IS |  |  |  |  |  |  |  | ○ | ○ |  |
|  |  | J17-106C | *B. nipponica* | IS |  |  |  |  |  |  |  | ○ | ○ |  |
|  |  | J17-106E | *B. nipponica* | IS |  |  |  |  |  |  |  | ○ | ○ |  |
|  | J17-108 | J17-108 | *B. nipponica* | IS | Nagura Dam, Ishigaki, Okinawa, Japan | h4 |  | ○ | ○ |  |  | ○ | ○ | 2017.04.23 |
|  | J17-286 | J17-286 | *B. nipponica* | IS | Omoto Tunnel, Ishigaki, Okinawa, Japan | h4 |  | ○ | ○ |  |  | ○ | ○ | 2017.04.28 |
|  | J17-290 | J17-290B | *B. nipponica* | IS | Omoto Tunnel, Ishigaki, Okinawa, Japan | h4 |  | ○ | ○ |  |  | ○ | ○ | 2017.04.28 |
| Sub-total | 39 | 45 |  | IS |  | 14 |  | 5 | 38 | 3 | 2 | 43 | 42 |  |
|  | Iri-023 | su023 |  | IR | Iriomote, Okinawa, Japan | h4 |  |  | ○ | ● |  | ● |  | 2002.02.25 |
|  | Iri60 | su060 |  | IR | Funaura, Iriomote, Okinawa, Japan | h4 |  |  | ○ | ● |  | ● |  | 2002.03.29 |
|  | J09-118 |  |  | IR | Urauti, Iriomote, Okinawa, Japan |  |  |  | ○ |  |  |  |  | 2009.10.06 |
|  | J09-119 |  |  | IR | Komi, Iriomote, Okinawa, Japan |  |  |  | ○ |  |  |  |  | 2009.10.06 |
|  | J09-120 | J09-120A | *B. nipponica* | IR | Komi, Iriomote, Okinawa, Japan |  |  |  | ○ |  |  | ○ | ○ | 2009.10.06 |
|  | J09-121 | J09-121A | *B. nipponica* | IR | Komi, Iriomote, Okinawa, Japan |  |  |  | ○ |  |  | ○ | ○ | 2009.10.06 |
|  |  | J09-121C | *B. nipponica* | IR |  |  |  |  |  |  |  | ○ | ○ |  |
|  |  | J09-121D | *B. nipponica* | IR |  |  |  |  |  |  |  |  | ○ |  |
|  |  | J09-121mA | *B. nipponica* | IR |  |  |  |  |  |  |  |  | ○ |  |
|  |  | J09-121mB | *B. nipponica* | IR |  |  |  |  |  |  |  | ○ | ○ |  |
|  |  | J09-121mC | *B. nipponica* | IR |  |  |  |  |  |  |  | ○ | ○ |  |
|  | J09-122 |  |  | IR | Komi, Iriomote, Okinawa, Japan |  |  |  | ○ |  |  |  |  | 2009.10.06 |
|  | J09-123 |  |  | IR | Komi, Iriomote, Okinawa, Japan |  |  |  | ○ |  |  |  |  | 2009.10.06 |
|  | J09-124 |  |  | IR | Ootomi, Iriomote, Okinawa, Japan |  |  |  | ○ |  |  |  |  | 2009.10.06 |
|  | J09-125 |  |  | IR | Ootomi, Iriomote, Okinawa, Japan |  |  |  | ○ |  |  |  |  | 2009.10.06 |
|  | J09-126 |  |  | IR | Komi, Iriomote, Okinawa, Japan |  |  |  | ○ |  |  |  |  | 2009.10.06 |
|  | J09-127 |  |  | IR | Mihara, Iriomote, Okinawa, Japan |  |  |  | ○ |  |  |  |  | 2009.10.06 |
|  | J09-128 | J09-128A |  | IR | Oomijya River, Iriomote, Okinawa, Japan |  |  |  | ○ |  |  | ○ |  | 2009.10.06 |
|  |  | J09-128B |  | IR |  |  |  |  |  |  |  | ○ |  |  |
|  | J09-129 |  |  | IR | Nakano, Iriomote, Okinawa, Japan |  |  |  | ○ |  |  |  |  | 2009.10.07 |
|  | J09-130 |  |  | IR | Hoshidate, Iriomote, Okinawa, Japan |  |  |  | ○ |  |  |  |  | 2009.10.07 |
|  | J09-131 |  |  | IR | Sonai, Iriomote, Okinawa, Japan |  |  |  | ○ |  |  |  |  | 2009.10.07 |
|  | J17-223 |  |  | IR | Komi, Iriomote, Okinawa, Japan | h4 |  |  | ○ |  |  |  |  | 2017.04.26 |
|  | J17-224 |  |  | IR | Komi, Iriomote, Okinawa, Japan | h4 |  |  | ○ |  |  |  |  | 2017.04.26 |
|  | J17-225 |  |  | IR | Komi, Iriomote, Okinawa, Japan | h4 |  |  | ○ |  |  |  |  | 2017.04.26 |
|  | J17-226 |  |  | IR | Komi, Iriomote, Okinawa, Japan | h4 |  |  | ○ |  |  |  |  | 2017.04.26 |
|  | J17-231 |  |  | IR | Funaura, Iriomote, Okinawa, Japan | h4 |  |  | ○ |  |  |  |  | 2017.04.26 |
|  | J17-233 |  |  | IR | Funaura, Iriomote, Okinawa, Japan | h4 |  |  | ○ |  |  |  |  | 2017.04.26 |
|  | J17-234 |  |  | IR | Funaura, Iriomote, Okinawa, Japan | h4 |  |  | ○ |  |  |  |  | 2017.04.26 |
|  | J17-238 |  |  | IR | Funaura, Iriomote, Okinawa, Japan | h4 |  |  | ○ |  |  |  |  | 2017.04.26 |
|  | J17-242 |  |  | IR | Ndara River, Iriomote, Okinawa, Japan | h4 |  |  | ○ |  |  |  |  | 2017.04.26 |
|  | J17-243 | J17-243mA | *B. nipponica* | IR | Ndara River, Iriomote, Okinawa, Japan | h4 |  |  | ○ |  |  | ○ | ○ | 2017.04.26 |
|  |  | J17-243mB | *B. nipponica* | IR |  |  |  |  |  |  |  | ○ | ○ |  |
|  | J17-245 |  |  | IR | Ndara River, Iriomote, Okinawa, Japan | h4 |  |  | ○ |  |  |  |  | 2017.04.26 |
|  | J17-246 |  |  | IR | Ndara River, Iriomote, Okinawa, Japan | h4 |  |  | ○ |  |  |  |  | 2017.04.26 |
|  | J17-250 | J17-250 | *B. nipponica* | IR | Ndara River, Iriomote, Okinawa, Japan | h4 |  |  | ○ |  |  | ○ | ○ | 2017.04.26 |
|  | J17-256 | J17-256mA | *B. nipponica* | IR | Oomijya River, Iriomote, Okinawa, Japan | h4 |  |  | ○ |  |  | ○ | ○ | 2017.04.26 |
|  | J17-266 | J17-266A | *B. nipponica* | IR | Takana, Iriomote, Okinawa, Japan | h4 |  |  | ○ |  |  | ○ | ○ | 2017.04.27 |
|  |  | J17-266B | *B. nipponica* | IR |  |  |  |  |  |  |  | ○ | ○ |  |
|  | J17-267 |  |  | IR | Takana, Iriomote, Okinawa, Japan | h4 |  |  | ○ |  |  |  |  | 2017.04.27 |
|  | J17-274 |  |  | IR | Iriomote, Okinawa, Japan | h4 |  |  | ○ |  |  |  |  | 2017.04.27 |
|  | J17-276 | J17-276 | *B. nipponica* | IR | Iriomote, Okinawa, Japan | h4 |  |  | ○ |  |  | ○ | ○ | 2017.04.27 |
|  | J17-281 |  |  | IR | Urauti River, Iriomote, Okinawa, Japan | h4 |  |  | ○ |  |  |  |  | 2017.04.27 |
|  | J17-282 |  |  | IR | Urauti River, Iriomote, Okinawa, Japan | h4 |  |  | ○ |  |  |  |  | 2017.04.27 |
| Sub-total | 36 | 18 |  | IR |  | 22 |  |  | 36 | 2 |  | 16 | 14 |  |
|  | Yona64 | su064 | *B. nipponica* | YN | Mt. Urabu-dake, Yonaguni, Okinawa, Japan | h5 |  |  | ○ | ● |  | ● |  | 2002.06.01 |
|  | J17-119 | J17-119mA | *B. nipponica* | YN | Yonaguni, Okinawa, Japan | h5 |  |  | ○ |  |  | ○ | ○ | 2017.04.24 |
|  |  | J17-119mB | *B. nipponica* | YN |  |  |  |  |  |  |  | ○ | ○ |  |
|  | J17-120 |  |  | YN | Yonaguni, Okinawa, Japan | h5 |  |  | ○ |  |  |  |  | 2017.04.24 |
|  | J17-123 |  |  | YN | Mt. Kubura-dake, Yonaguni, Okinawa, Japan | h5 |  |  | ○ |  |  |  |  | 2017.04.24 |
|  | J17-124 |  |  | YN | Mt. Kubura-dake, Yonaguni, Okinawa, Japan | h5 |  |  | ○ |  |  |  |  | 2017.04.24 |
|  | J17-125 |  |  | YN | Mt. Kubura-dake, Yonaguni, Okinawa, Japan | h5 |  |  | ○ |  |  |  |  | 2017.04.24 |
|  | J17-127 |  |  | YN | Mt. Kubura-dake, Yonaguni, Okinawa, Japan | h5 |  |  | ○ |  |  |  |  | 2017.04.24 |
|  | J17-128 |  |  | YN | Mt. Kubura-dake, Yonaguni, Okinawa, Japan | h5 |  |  | ○ |  |  |  |  | 2017.04.24 |
|  | J17-132 |  |  | YN | Mt. Kubura-dake, Yonaguni, Okinawa, Japan | h5 |  |  | ○ |  |  |  |  | 2017.04.24 |
|  | J17-137 | J17-137mA | *B. nipponica* | YN | Mt. Kubura-dake, Yonaguni, Okinawa, Japan | h5 |  |  | ○ |  |  | ○ | ○ | 2017.04.24 |
|  |  | J17-137mB | *B. nipponica* | YN |  |  |  |  |  |  |  | ○ | ○ |  |
|  |  | J17-137mC | *B. nipponica* | YN |  |  |  |  |  |  |  | ○ | ○ |  |
|  |  | J17-137mD | *B. nipponica* | YN |  |  |  |  |  |  |  | ○ | ○ |  |
|  |  | J17-137mE | *B. nipponica* | YN |  |  |  |  |  |  |  | ○ | ○ |  |
|  | J17-138 |  |  | YN | Mt. Kubura-dake, Yonaguni, Okinawa, Japan | h5 |  |  | ○ |  |  |  |  | 2017.04.24 |
|  | J17-140 |  |  | YN | Mt. Kubura-dake, Yonaguni, Okinawa, Japan | h5 |  |  | ○ |  |  |  |  | 2017.04.24 |
|  | J17-142 |  |  | YN | Mt. Kubura-dake, Yonaguni, Okinawa, Japan | h5 |  |  | ○ |  |  |  |  | 2017.04.24 |
|  | J17-143 |  |  | YN | Mt. Kubura-dake, Yonaguni, Okinawa, Japan | h5 |  |  | ○ |  |  |  |  | 2017.04.24 |
|  | J17-146 |  |  | YN | Mt. Kubura-dake, Yonaguni, Okinawa, Japan | h5 |  |  | ○ |  |  |  |  | 2017.04.24 |
|  | J17-147 |  |  | YN | Yonaguni, Okinawa, Japan | h5 |  |  | ○ |  |  |  |  | 2017.04.24 |
|  | J17-149 |  |  | YN | Yonaguni, Okinawa, Japan | h5 |  |  | ○ |  |  |  |  | 2017.04.24 |
|  | J17-151 |  |  | YN | Yonaguni, Okinawa, Japan | h5 |  |  | ○ |  |  |  |  | 2017.04.24 |
|  | J17-152 |  |  | YN | Yonaguni, Okinawa, Japan | h5 |  |  | ○ |  |  |  |  | 2017.04.24 |
|  | J17-153 |  |  | YN | Mt. Inbi-dake, Yonaguni, Okinawa, Japan | h5 |  |  | ○ |  |  |  |  | 2017.04.24 |
|  | J17-154 |  |  | YN | Mt. Inbi-dake, Yonaguni, Okinawa, Japan | h5 |  |  | ○ |  |  |  |  | 2017.04.24 |
|  | J17-155 | J17-155mA | *B. nipponica* | YN | Yonaguni, Okinawa, Japan | h5 |  |  | ○ |  |  | ○ | ○ | 2017.04.24 |
|  | J17-156 | J17-156mA | *B. nipponica* | YN | Yonaguni, Okinawa, Japan | h5 |  |  | ○ |  |  | ○ | ○ | 2017.04.24 |
|  |  | J17-156mB | *B. nipponica* | YN |  |  |  |  |  |  |  | ○ | ○ |  |
|  |  | J17-156mC | *B. nipponica* | YN |  |  |  |  |  |  |  | ○ | ○ |  |
|  |  | J17-156mE | *B. nipponica* | YN |  |  |  |  |  |  |  | ○ | ○ |  |
|  | J17-159 |  |  | YN | Yonaguni, Okinawa, Japan | h5 |  |  | ○ |  |  |  |  | 2017.04.25 |
|  | J17-167 |  |  | YN | Yonaguni, Okinawa, Japan | h5 |  |  | ○ |  |  |  |  | 2017.04.25 |
|  | J17-168 |  |  | YN | Yonaguni, Okinawa, Japan | h5 |  |  | ○ |  |  |  |  | 2017.04.25 |
|  | J17-175 | J17-175mA | *B. nipponica* | YN | Yonaguni, Okinawa, Japan | h5 |  |  | ○ |  |  | ○ | ○ | 2017.04.25 |
|  | J17-176 | J17-176mA | *B. nipponica* | YN | Yonaguni, Okinawa, Japan | h5 |  |  | ○ |  |  | ○ | ○ | 2017.04.25 |
|  | J17-179 |  |  | YN | Yonaguni, Okinawa, Japan | h5 |  |  | ○ |  |  |  |  | 2017.04.25 |
|  | J17-181 | J17-181mA | *B. nipponica* | YN | Yonaguni, Okinawa, Japan | h5 |  |  | ○ |  |  | ○ | ○ | 2017.04.25 |
|  | J17-182 | J17-182A | *B. nipponica* | YN | Yonaguni, Okinawa, Japan | h5 |  |  | ○ |  |  | ○ | ○ | 2017.04.25 |
|  |  | J17-182B | *B. nipponica* | YN |  |  |  |  |  |  |  | ○ | ○ |  |
|  |  | J17-182C | *B. nipponica* | YN |  |  |  |  |  |  |  | ○ | ○ |  |
|  | J17-186 | J17-186A | *B. nipponica* | YN | Yonaguni, Okinawa, Japan | h5 |  |  | ○ |  |  | ○ | ○ | 2017.04.25 |
|  |  | J17-186B | *B. nipponica* | YN |  |  |  |  |  |  |  | ○ | ○ |  |
|  | J17-187 | J17-187A | *B. nipponica* | YN | Yonaguni, Okinawa, Japan | h5 |  |  | ○ |  |  | ○ | ○ | 2017.04.25 |
|  |  | J17-187B | *B. nipponica* | YN |  |  |  |  |  |  |  | ○ | ○ |  |
|  | J17-190 | J17-190 | *B. nipponica* | YN | Yonaguni, Okinawa, Japan | h5 |  |  | ○ |  |  | ○ | ○ | 2017.04.25 |
|  | J17-191 | J17-191A | *B. nipponica* | YN | Yonaguni, Okinawa, Japan | h4 |  |  | ○ |  |  | ○ | ○ | 2017.04.25 |
|  |  | J17-191B | *B. nipponica* | YN |  |  |  |  |  |  |  | ○ | ○ |  |
| Sub-total | 35 | 26 |  | YN |  | 35 |  |  | 35 | 1 |  | 26 | 25 |  |
|  | TW003 |  |  | TW | Bitou Cape, Jilong, Taiwain | h6 |  |  | ● |  |  |  |  | 2008.09.03 |
|  | TW007 |  |  | TW | Bitou Cape, Jilong, Taiwain | h6 |  |  | ● |  |  |  |  | 2008.09.03 |
|  | TW015 |  |  | TW | Tianhxiyuan, Taipei, Taiwan | h6 |  |  | ● |  |  |  |  | 2008.09.05 |
|  | TW016 |  |  | TW | Tianhxiyuan, Taipei, Taiwan | h6 |  |  | ● |  |  |  |  | 2008.09.05 |
|  | TW018 | TW018 | *B. nipponica* | TW | Xiaoyoukeng, Taipei, Taiwan | h7 |  |  | ● | ● |  | ● | ● | 2008.09.05 |
|  | TW019 |  |  | TW | Xiaoyoukeng, Taipei, Taiwan | h7 |  |  | ● |  |  |  |  | 2008.09.05 |
|  | TW020 |  |  | TW | Xiaoyoukeng, Taipei, Taiwan | h7 |  |  | ● |  |  |  |  | 2008.09.05 |
|  | TW041 | TW041 | *B. nipponica* | TW | Kenting, Pingdong, Taiwan | h7 |  |  | ● | ● |  | ● | ● | 2008.09.08 |
|  | TW060 |  |  | TW | Jufu, Taidong, Taiwan | h8 |  |  | ● |  |  |  |  | 2008.09.10 |
|  | TW063 | TW063 | *B. nipponica* | TW | Jufu, Taidong, Taiwan | h8 |  |  | ● | ● |  | ● | ● | 2008.09.10 |
|  | TW11-001 | TW011-01-1 | *B. nipponica* | TW | Guoxing, Nantou, Taiwan | h9 |  |  | ● |  |  |  |  | 2011.09.06 |
|  | TW11-002 | TW011-02-1 | *B. nipponica* | TW | Guoxing, Nantou, Taiwan | h9 |  |  | ● |  |  | ● | ● | 2011.09.06 |
|  | TW11-023 | TW011-23-1 | *B. nipponica* | TW | Shuili, Nantou, Taiwan | h10 |  |  | ● |  |  |  | ● | 2011.09.06 |
|  |  | TW011-23-3 | *B. nipponica* | TW |  |  |  |  |  |  |  |  | ● |  |
|  | TW11-024 |  |  | TW | Shuili, Nantou, Taiwan | h9 |  |  | ● |  |  |  |  | 2011.09.06 |
|  | TW11-025 |  |  | TW | Shuili, Nantou, Taiwan | h9 |  |  | ● |  |  |  |  | 2011.09.06 |
|  | TW11-026 |  |  | TW | Shuili, Nantou, Taiwan | h9 |  |  | ● |  |  |  |  | 2011.09.06 |
|  | TW11-027 |  |  | TW | Shuili, Nantou, Taiwan | h9 |  |  | ● |  |  |  |  | 2011.09.06 |
|  | TW11-031 |  |  | TW | Shuili, Nantou, Taiwan | h10 |  |  | ● |  |  |  |  | 2011.09.06 |
|  | TW11-039 | TW11-39-1 | *B. nipponica* | TW | Shuili, Nantou, Taiwan | h11 |  | ○ | ● |  |  |  | ● | 2011.09.06 |
|  | TW11-095 | TW11-95-1 | *B. nipponica* | TW | Kenting, Pingdong, Taiwan | h12 |  |  | ● |  |  |  | ● | 2011.09.09 |
|  |  | TW11-95-2 | *B. nipponica* | TW |  |  |  |  |  |  |  | ● | ● |  |
|  | TW11-210 |  |  | TW | Fushan Botanical Garden, Yuanshan, Yilan, Taiwan | h6 |  |  | ● |  |  |  |  | 2011.09.16 |
|  | TW11-211 |  |  | TW | Fushan Botanical Garden, Yuanshan, Yilan, Taiwan | h6 |  |  | ● |  |  |  |  | 2011.09.16 |
|  | TW11-212 |  |  | TW | Fushan Botanical Garden, Yuanshan, Yilan, Taiwan | h6 |  |  | ● |  |  |  |  | 2011.09.16 |
|  | TW11-224 |  |  | TW | Gongliao, Xinbei, Taiwan | h8 |  |  | ● |  |  |  |  | 2011.09.17 |
|  | TW11-225 |  |  | TW | Gongliao, Xinbei, Taiwan | h8 |  |  | ● |  |  |  |  | 2011.09.17 |
|  | TW14-014 | TW14-014-A | *B. nipponica* | TW | Mudan, Pingdong, Taiwan |  |  |  |  | ○ |  |  | ○ | 2014.03.22 |
|  |  | TW14-014-b | *B. nipponica* | TW |  |  |  |  |  | ○ |  |  | ○ |  |
|  |  | TW14-014-c | *B. nipponica* | TW |  |  |  |  |  | ○ |  |  | ○ |  |
|  |  | TW14-014-d | *B. nipponica* | TW |  |  |  |  |  | ○ |  |  | ○ |  |
|  |  | TW14-014-e | *B. nipponica* | TW |  |  |  |  |  | ○ |  |  | ○ |  |
|  | TW14-015 | TW14-015 | *B. nipponica* | TW | Mudan, Pingdong, Taiwan |  |  |  |  | ○ |  |  | ○ | 2014.03.22 |
|  | TW14-032 | TW14-032-a1 | *B. nipponica* | TW | Guoxing, Nantou, Taiwan |  |  |  |  | ○ |  |  | ○ | 2014.03.24 |
|  |  | TW14-032-a2 | *B. nipponica* | TW |  |  |  |  |  | ○ |  |  | ○ |  |
|  |  | TW14-032-b | *B. nipponica* | TW |  |  |  |  |  | ○ |  |  | ○ |  |
|  | TW14-033 | TW14-033-1a | *B. nipponica* | TW | Guoxing, Nantou, Taiwan |  |  |  |  | ○ |  |  | ○ | 2014.03.24 |
|  |  | TW14-033-2a | *B. nipponica* | TW |  |  |  |  |  | ○ |  | ○ | ○ |  |
|  |  | TW14-033-3a | *B. nipponica* | TW |  |  |  |  |  | ○ |  | ○ | ○ |  |
|  | TW14-035 | TW14-035-1a | *B. nipponica* | TW | Guoxing, Nantou, Taiwan |  |  |  |  | ○ |  | ○ | ○ | 2014.03.24 |
|  |  | TW14-035-2a | *B. nipponica* | TW |  |  |  |  |  | ○ |  |  | ○ |  |
|  |  | TW14-035-b | *B. nipponica* | TW |  |  |  |  |  | ○ |  |  | ○ |  |
|  | TW14-038 | TW14-038 | *B. nipponica* | TW | Guoxing, Nantou, Taiwan |  |  |  |  | ○ |  |  | ○ | 2014.03.24 |
|  | TW16-137 |  |  | TW | Shiding, Xinbei, Taiwan | h7 |  |  | ○ |  |  |  |  | 2016.08.10 |
|  | TW16-153 |  |  | TW | Shiding, Xinbei, Taiwan | h7 |  |  | ○ |  |  |  |  | 2016.08.10 |
|  | TW16-154 |  |  | TW | Shiding, Xinbei, Taiwan | h7 |  |  | ○ |  |  |  |  | 2016.08.10 |
|  | TW16-156 |  |  | TW | Gold Ecological Park, Shiding, Xinbei, Taiwan | h7 |  |  | ○ |  |  |  |  | 2016.08.11 |
|  | TW16-157 | TW16-157-A | *B. nipponica* | TW | Gold Ecological Park, Shiding, Xinbei, Taiwan | h7 |  |  | ○ | ○ |  |  | ○ | 2016.08.11 |
|  |  | TW16-157-B | *B. nipponica* | TW |  |  |  |  |  | ○ |  | ○ | ○ |  |
|  | TW16-158 | TW16-158 | *B. nipponica* | TW | Gold Ecological Park, Shiding, Xinbei, Taiwan |  |  |  |  | ○ |  |  | ○ | 2016.08.11 |
|  | TW16-159 |  |  | TW | Gold Ecological Park, Shiding, Xinbei, Taiwan | h7 |  |  | ○ |  |  |  |  | 2016.08.11 |
|  | TW16-161 | TW16-161-A | *B. nipponica* | TW | Shiding, Xinbei, Taiwan | h6 |  |  | ○ | ○ |  | ○ | ○ | 2016.08.11 |
|  |  | TW16-161-B | *B. nipponica* | TW |  |  |  |  |  | ○ |  |  | ○ |  |
|  |  | TW16-161-C | *B. nipponica* | TW |  |  |  |  |  | ○ |  | ○ | ○ |  |
|  | TW16-162 |  |  | TW | Shiding, Xinbei, Taiwan | h6 |  |  | ○ |  |  |  |  | 2016.08.11 |
|  | TW16-163 | TW16-163-A | *B. nipponica* | TW | Shiding, Xinbei, Taiwan |  |  |  |  | ○ | ○ | ○ | ○ | 2016.08.11 |
|  |  | TW16-163-B | *B. nipponica* | TW |  |  |  |  |  | ○ | ○ | ○ | ○ |  |
|  | TW16-164 | TW16-164-A | *B. nipponica* | TW | Shiding, Xinbei, Taiwan | h6 |  |  | ○ | ○ |  | ○ | ○ | 2016.08.11 |
|  |  | TW16-164-B | *B. nipponica* | TW |  |  |  |  |  | ○ |  | ○ | ○ |  |
|  |  | TW16-164-C | *B. nipponica* | TW |  |  |  |  |  | ○ |  | ○ | ○ |  |
|  | TW16-187 |  |  | TW | Guishandao, Toucheng, Yilan, Taiwan | h7 |  |  | ○ |  |  |  |  | 2016.08.12 |
|  | TW16-193 |  |  | TW | Guishandao, Toucheng, Yilan, Taiwan | h7 |  |  | ○ |  |  |  |  | 2016.08.12 |
|  | TW16-202 |  |  | TW | Guishandao, Toucheng, Yilan, Taiwan | h7 |  |  | ○ |  |  |  |  | 2016.08.12 |
|  | TW16-213 |  |  | TW | Guishandao, Toucheng, Yilan, Taiwan | h7 |  |  | ○ |  |  |  |  | 2016.08.12 |
|  | TW16-253 |  |  | TW | Guishandao, Toucheng, Yilan, Taiwan | h7 |  |  | ○ |  |  |  |  | 2016.08.12 |
|  | TW16-316 |  |  | TW | Nanao, Yilan, Taiwan | h6 |  |  | ○ |  |  |  |  | 2016.08.14 |
| Sub-total | 48 | 37 |  | TW |  | 40 |  | 1 | 40 | 30 | 2 | 16 | 36 |  |
|  | TW11-130 |  |  | LY | Datienchih, Lanyu, Taiwan | h8 |  |  | ○ |  |  |  |  | 2011.09.11 |
|  | TW11-131 |  |  | LY | Datienchih, Lanyu, Taiwan | h8 |  |  | ○ |  |  |  |  | 2011.09.11 |
|  | TW11-132 |  |  | LY | Datienchih, Lanyu, Taiwan | h8 |  |  | ○ |  |  |  |  | 2011.09.11 |
|  | TW11-133 | TW11-133-1 | *B. nipponica* | LY | Datienchih, Lanyu, Taiwan | h8 |  | ○ | ○ |  |  | ● | ○ | 2011.09.11 |
|  |  | TW11-133-3 | *B. nipponica* | LY | Datienchih, Lanyu, Taiwan |  |  |  |  |  |  | ● | ○ |  |
|  | TW11-134 | TW11-134-1 | *B. nipponica* | LY | Datienchih, Lanyu, Taiwan | h8 |  | ○ | ○ |  |  |  | ○ | 2011.09.11 |
|  |  | TW11-134-2 | *B. nipponica* | LY | Datienchih, Lanyu, Taiwan |  |  |  |  |  |  |  | ○ |  |
|  | TW11-135 |  |  | LY | Datienchih, Lanyu, Taiwan | h8 |  |  | ○ |  |  |  |  | 2011.09.11 |
|  | TW11-136 | TW11-136-1 | *B. nipponica* | LY | Datienchih, Lanyu, Taiwan | h8 |  | ○ | ○ |  |  | ● | ○ | 2011.09.11 |
|  | TW11-137 |  |  | LY | Datienchih, Lanyu, Taiwan | h8 |  |  | ○ |  |  |  |  | 2011.09.11 |
|  | TW13-055 | TW13-055 | *B. nipponica* | LY | Datienchih, Lanyu, Taiwan |  |  |  |  |  |  | ● | ○ | 2013.03.21 |
|  | TW13-056 | TW13-056 | *B. nipponica* | LY | Datienchih, Lanyu, Taiwan |  |  |  |  |  |  | ● | ○ | 2013.03.22 |
|  | TW13-057 | TW13-057 | *B. nipponica* | LY | Datienchih, Lanyu, Taiwan |  |  |  |  |  |  | ● | ○ | 2013.03.22 |
| Sub-total | 11 | 8 |  | LY |  | 8 |  | 3 | 8 |  |  | 6 | 8 |  |
|  | MS13-001 |  |  | MT | Nangan, Lienchiang, Matsu, Taiwan | h13 |  |  | ● |  |  |  |  | 2013.08.09 |
|  | MS13-002 |  |  | MT | Nangan, Lienchiang, Matsu, Taiwan | h13 |  |  | ● |  |  |  |  | 2013.08.09 |
|  | MS13-003 |  |  | MT | Nangan, Lienchiang, Matsu, Taiwan | h13 |  |  | ● |  |  |  |  | 2013.08.09 |
|  | MS13-004 |  |  | MT | Nangan, Lienchiang, Matsu, Taiwan | h13 |  |  | ● |  |  |  |  | 2013.08.09 |
|  | MS13-005 | MS13-005 | *B. nipponica* | MT | Nangan, Lienchiang, Matsu, Taiwan | h13 |  |  | ● |  |  | ● | ● | 2013.08.10 |
|  | MS13-006 |  |  | MT | Nangan, Lienchiang, Matsu, Taiwan | h13 |  |  | ● |  |  |  |  | 2013.08.10 |
|  | MS13-007 |  |  | MT | Nangan, Lienchiang, Matsu, Taiwan | h13 |  |  | ● |  |  |  |  | 2013.08.10 |
|  | MS13-008 |  |  | MT | Nangan, Lienchiang, Matsu, Taiwan | h13 |  |  | ● |  |  |  |  | 2013.08.10 |
|  | MS13-009 |  |  | MT | Nangan, Lienchiang, Matsu, Taiwan | h13 |  |  | ● |  |  |  |  | 2013.08.10 |
|  | MS13-010 |  |  | MT | Nangan, Lienchiang, Matsu, Taiwan | h13 |  |  | ● |  |  |  |  | 2013.08.10 |
|  | MS13-011 | MS13-011b | *B. nipponica* | MT | Nangan, Lienchiang, Matsu, Taiwan | h13 |  |  | ● |  |  | ● | ● | 2013.08.10 |
|  | MS13-012 |  |  | MT | Nangan, Lienchiang, Matsu, Taiwan | h13 |  |  | ● |  |  |  |  | 2013.08.10 |
|  | MS13-013 |  |  | MT | Nangan, Lienchiang, Matsu, Taiwan | h13 |  |  | ● |  |  |  |  | 2013.08.10 |
|  | MS13-014 |  |  | MT | Nangan, Lienchiang, Matsu, Taiwan | h13 |  |  | ● |  |  |  |  | 2013.08.10 |
|  | MS13-016 | MS13-016a | *B. nipponica* | MT | Beigan, Lienchiang, Matsu, Taiwan | h13 |  |  | ● |  |  |  | ● | 2013.08.10 |
|  | MS13-019 |  |  | MT | Beigan, Lienchiang, Matsu, Taiwan | h13 |  |  | ● |  |  |  |  | 2013.08.10 |
|  | MS13-020 | MS13-020a | *B. nipponica* | MT | Beigan, Lienchiang, Matsu, Taiwan | h13 |  |  | ● |  |  |  | ● | 2013.08.11 |
|  |  | MS13-020b | *B. nipponica* | MT |  |  |  |  |  |  |  |  | ● |  |
|  |  | MS13-020c | *B. nipponica* | MT |  |  |  |  |  |  |  |  | ● |  |
|  | MS13-021 |  |  | MT | Beigan, Lienchiang, Matsu, Taiwan | h13 |  |  | ● |  |  |  |  | 2013.08.11 |
|  | MS13-022 |  |  | MT | Beigan, Lienchiang, Matsu, Taiwan | h13 |  |  | ● |  |  |  |  | 2013.08.11 |
|  | MS13-023 |  |  | MT | Beigan, Lienchiang, Matsu, Taiwan | h13 |  |  | ● |  |  |  |  | 2013.08.11 |
|  | MS13-025 | MS13-025c | *B. nipponica* | MT | Beigan, Lienchiang, Matsu, Taiwan | h13 |  |  | ● |  |  | ● | ● | 2013.08.11 |
|  | MS13-026 |  |  | MT | Beigan, Lienchiang, Matsu, Taiwan | h13 |  |  | ● |  |  |  |  | 2013.08.11 |
|  | MS13-027 | MS13-027 | *B. nipponica* | MT | Beigan, Lienchiang, Matsu, Taiwan | h13 |  |  | ● |  |  |  | ● | 2013.08.11 |
|  | MS13-029 |  |  | MT | Beigan, Lienchiang, Matsu, Taiwan | h13 |  |  | ● |  |  |  |  | 2013.08.11 |
|  | MS13-030 |  |  | MT | Beigan, Lienchiang, Matsu, Taiwan | h13 |  |  | ● |  |  |  |  | 2013.08.11 |
|  | MS13-031 |  |  | MT | Beigan, Lienchiang, Matsu, Taiwan | h13 |  |  | ● |  |  |  |  | 2013.08.11 |
|  | MS13-032 |  |  | MT | Beigan, Lienchiang, Matsu, Taiwan | h13 |  |  | ● |  |  |  |  | 2013.08.11 |
|  | MS13-033 | MS13-033a | *B. nipponica* | MT | East Juguang, Lienchiang, Matsu, Taiwan | h13 |  |  | ● |  |  |  | ● | 2013.08.11 |
|  |  | MS13-033b | *B. nipponica* | MT |  |  |  |  |  |  |  |  | ● |  |
|  |  | MS13-033c | *B. nipponica* | MT |  |  |  |  |  |  |  | ● | ● |  |
|  | MS13-034 |  |  | MT | East Juguang, Lienchiang, Matsu, Taiwan | h13 |  |  | ● |  |  |  |  | 2013.08.12 |
|  | MS13-035 | MS13-035a | *B. nipponica* | MT | East Juguang, Lienchiang, Matsu, Taiwan | h13 |  |  | ● |  |  |  | ● | 2013.08.11 |
|  |  | MS13-035b | *B. nipponica* | MT |  |  |  |  |  |  |  |  | ● |  |
|  | MS13-036 | MS13-036b | *B. nipponica* | MT | East Juguang, Lienchiang, Matsu, Taiwan | h13 |  |  | ● |  |  | ● | ● | 2013.08.11 |
|  | MS13-037 |  |  | MT | East Juguang, Lienchiang, Matsu, Taiwan | h13 |  |  | ● |  |  |  |  | 2013.08.12 |
|  | MS13-038 |  |  | MT | East Juguang, Lienchiang, Matsu, Taiwan | h13 |  |  | ● |  |  |  |  | 2013.08.12 |
|  | MS13-039 |  |  | MT | East Juguang, Lienchiang, Matsu, Taiwan | h13 |  |  | ● |  |  |  |  | 2013.08.11 |
|  | MS13-040 | MS13-040a | *B. nipponica* | MT | East Juguang, Lienchiang, Matsu, Taiwan | h13 |  |  | ● |  |  | ● | ● | 2013.08.11 |
|  |  | MS13-040b | *B. nipponica* | MT |  |  |  |  |  |  |  |  | ● |  |
|  |  | MS13-040c | *B. nipponica* | MT |  |  |  |  |  |  |  | ● | ● |  |
|  |  | MS13-040RNA | *B. nipponica* | MT |  |  |  |  |  |  |  | ● | ● |  |
|  | MS13-041 |  |  | MT | East Juguang, Lienchiang, Matsu, Taiwan | h13 |  |  | ● |  |  |  |  | 2013.08.11 |
|  | MS13-042 | MS13-042b | *B. nipponica* | MT | East Juguang, Lienchiang, Matsu, Taiwan | h13 |  |  | ● |  |  | ● | ● | 2013.08.12 |
|  |  | MS13-042c | *B. nipponica* | MT |  |  |  |  |  |  |  | ● | ● |  |
|  | MS13-043 | MS13-043b | *B. nipponica* | MT | East Juguang, Lienchiang, Matsu, Taiwan | h13 |  |  | ● |  |  | ● | ● | 2013.08.11 |
|  | MS13-044 |  |  | MT | East Juguang, Lienchiang, Matsu, Taiwan | h13 |  |  | ● |  |  |  |  | 2013.08.12 |
|  | MS13-045 |  |  | MT | East Juguang, Lienchiang, Matsu, Taiwan | h13 |  |  | ● |  |  |  |  | 2013.08.12 |
|  | MS13-046 | MS13-046a | *B. nipponica* | MT | West Juguang, Lienchiang, Matsu, Taiwan | h13 |  |  | ● |  |  |  | ● | 2013.08.12 |
|  |  | MS13-046b | *B. nipponica* | MT |  |  |  |  |  |  |  | ● | ● |  |
|  |  | MS13-046c | *B. nipponica* | MT |  |  |  |  |  |  |  |  | ● |  |
|  | MS13-047 | MS13-047a | *B. nipponica* | MT | West Juguang, Lienchiang, Matsu, Taiwan | h13 |  |  | ● |  |  |  | ● | 2013.08.13 |
|  |  | MS13-047b | *B. nipponica* | MT |  |  |  |  |  |  |  |  | ● |  |
|  | MS13-048 | MS13-048a | *B. nipponica* | MT | West Juguang, Lienchiang, Matsu, Taiwan | h13 |  |  | ● |  |  | ● | ● | 2013.08.12 |
|  |  | MS13-048b | *B. nipponica* | MT |  |  |  |  |  |  |  |  | ● |  |
|  |  | MS13-048d | *B. nipponica* | MT |  |  |  |  |  |  |  |  | ● |  |
|  | MS13-049 | MS13-049a | *B. nipponica* | MT | West Juguang, Lienchiang, Matsu, Taiwan | h13 |  |  | ● |  |  | ● | ● | 2013.08.12 |
|  |  | MS13-049b | *B. nipponica* | MT |  |  |  |  |  |  |  |  | ● |  |
|  |  | MS13-049c | *B. nipponica* | MT |  |  |  |  |  |  |  | ● | ● |  |
|  | MS13-050 |  |  | MT | West Juguang, Lienchiang, Matsu, Taiwan | h13 |  |  | ● |  |  |  |  | 2013.08.13 |
|  | MS13-051 |  |  | MT | West Juguang, Lienchiang, Matsu, Taiwan | h13 |  |  | ● |  |  |  |  | 2013.08.13 |
|  | MS13-052 |  |  | MT | West Juguang, Lienchiang, Matsu, Taiwan | h13 |  |  | ● |  |  |  |  | 2013.08.13 |
|  | MS13-053 | MS13-053a | *B. nipponica* | MT | West Juguang, Lienchiang, Matsu, Taiwan |  |  |  |  |  |  |  | ● | 2013.08.12 |
|  |  | MS13-053b | *B. nipponica* | MT |  |  |  |  |  |  |  | ● | ● |  |
|  |  | MS13-053c | *B. nipponica* | MT |  |  |  |  |  |  |  | ● | ● |  |
|  | MS13-054 | MS13-054b | *B. nipponica* | MT | West Juguang, Lienchiang, Matsu, Taiwan | h13 |  |  | ● |  |  |  | ● | 2013.08.12 |
|  |  | MS13-054c | *B. nipponica* | MT |  |  |  |  |  |  |  | ● | ● |  |
|  |  | MS13-054d | *B. nipponica* | MT |  |  |  |  |  |  |  |  | ● |  |
|  |  | MS13-054e | *B. nipponica* | MT |  |  |  |  |  |  |  | ● | ● |  |
|  | MS13-055 | MS13-055b | *B. nipponica* | MT | West Juguang, Lienchiang, Matsu, Taiwan | h13 |  |  | ● |  |  | ● | ● | 2013.08.12 |
|  |  | MS13-055c | *B. nipponica* | MT |  |  |  |  |  |  |  |  | ● |  |
|  |  | MS13-055d | *B. nipponica* | MT |  |  |  |  |  |  |  |  | ● |  |
|  | MS13-056 | MS13-056a | *B. nipponica* | MT | West Juguang, Lienchiang, Matsu, Taiwan | h13 |  |  | ● |  |  | ● | ● | 2013.08.12 |
|  |  | MS13-056b | *B. nipponica* | MT |  |  |  |  |  |  |  |  | ● |  |
|  | MS13-057 | MS13-057a | *B. nipponica* | MT | West Juguang, Lienchiang, Matsu, Taiwan | h13 |  |  | ● |  |  |  | ● | 2013.08.13 |
|  |  | MS13-057b | *B. nipponica* | MT |  |  |  |  |  |  |  |  | ● |  |
|  |  | MS13-057RNA | *B. nipponica* | MT |  |  |  |  |  |  |  | ● | ● |  |
| Sub-total | 52 | 47 |  | MT |  | 51 |  |  | 51 |  |  | 22 | 47 |  |
|  | C17-004 |  |  | FJ | Jiaocheng, Ningde, Fujian, China | h14 |  |  | ○ |  |  |  |  | 2017.08.12 |
|  | C17-007 |  |  | FJ | Jiaocheng, Ningde, Fujian, China | h15 |  |  | ○ |  |  |  |  | 2017.08.12 |
|  | C17-009 | C17-009B | *B. nipponica* | FJ | Jiaocheng, Ningde, Fujian, China | h15 |  |  | ○ | ○ |  | ○ | ○ | 2017.08.12 |
|  |  | C17-009D | *B. nipponica* | FJ |  |  |  |  |  | ○ |  | ○ | ○ |  |
|  |  | C17-009E | *B. nipponica* | FJ |  |  |  |  |  | ○ |  | ○ | ○ |  |
|  | C17-010 | C17-010A | *B. silvestriana* | FJ | Jiaocheng, Ningde, Fujian, China | h15 |  |  | ○ | ○ | ○ | ○ |  | 2017.08.12 |
|  |  | C17-010C | *B. nipponica* | FJ |  |  |  |  |  | ○ | ○ | ○ | ○ |  |
|  |  | C17-010E | *B. nipponica* | FJ |  |  |  |  |  | ○ | ○ | ○ | ○ |  |
|  | C17-011 |  |  | FJ | Jiaocheng, Ningde, Fujian, China | h15 |  |  | ○ |  |  |  |  | 2017.08.12 |
|  | C17-012 |  |  | FJ | Jiaocheng, Ningde, Fujian, China | h15 |  |  | ○ |  |  |  |  | 2017.08.12 |
|  | C17-013 | C17-013A | *B. nipponica* | FJ | Jiaocheng, Ningde, Fujian, China | h15 |  | ○ | ○ | ○ | ○ | ○ | ○ | 2017.08.12 |
|  |  | C17-013C | *B. nipponica* | FJ |  |  |  |  |  | ○ | ○ | ○ | ○ |  |
|  |  | C17-013D | *B. nipponica* | FJ |  |  |  |  |  | ○ |  | ○ | ○ |  |
|  |  | C17-013E | *B. nipponica* | FJ |  |  |  |  |  | ○ |  | ○ | ○ |  |
|  | C17-014 |  |  | FJ | Jiaocheng, Ningde, Fujian, China | h15 |  |  | ○ |  |  |  |  | 2017.08.12 |
|  | C17-015 |  |  | FJ | Jiaocheng, Ningde, Fujian, China | h15 |  |  | ○ |  |  |  |  | 2017.08.12 |
|  | C17-016 |  |  | FJ | Jiaocheng, Ningde, Fujian, China | h15 |  |  | ○ |  |  |  |  | 2017.08.12 |
|  | C17-017 |  |  | FJ | Jiaocheng, Ningde, Fujian, China | h16 |  |  | ○ |  |  |  |  | 2017.08.12 |
|  | C17-018 |  |  | FJ | Jiaocheng, Ningde, Fujian, China | h14 |  |  | ○ |  |  |  |  | 2017.08.12 |
|  | C17-019 |  |  | FJ | Fuan, Ningde, Fujian, China | h15 |  | ○ | ○ |  |  |  |  | 2017.08.12 |
|  | C17-021 |  |  | FJ | Fuan, Ningde, Fujian, China | h15 |  | ○ | ○ |  |  |  |  | 2017.08.12 |
|  | C17-024 |  |  | FJ | Fuan, Ningde, Fujian, China | h15 |  |  | ○ |  |  |  |  | 2017.08.12 |
|  | C17-025 |  |  | FJ | Fuan, Ningde, Fujian, China | h17 |  |  | ○ |  |  |  |  | 2017.08.12 |
|  | C17-026 |  |  | FJ | Fuan, Ningde, Fujian, China | h15 |  |  | ○ |  |  |  |  | 2017.08.12 |
|  | C17-027 | C17-027A | *B. nipponica* | FJ | Fuan, Ningde, Fujian, China | h15 |  | ○ | ○ | ○ |  | ○ | ○ | 2017.08.12 |
|  |  | C17-027B | *B. nipponica* | FJ |  |  |  |  |  | ○ |  | ○ | ○ |  |
|  |  | C17-027C | *B. nipponica* | FJ |  |  |  |  |  | ○ |  | ○ | ○ |  |
|  |  | C17-027D | *B. nipponica* | FJ |  |  |  |  |  | ○ |  | ○ | ○ |  |
|  |  | C17-027E | *B. nipponica* | FJ |  |  |  |  |  | ○ |  | ○ | ○ |  |
|  | C17-028 |  |  | FJ | Fuan, Ningde, Fujian, China | h15 |  |  | ○ |  |  |  |  | 2017.08.12 |
|  | C17-029 |  |  | FJ | Fuan, Ningde, Fujian, China | h15 |  |  | ○ |  |  |  |  | 2017.08.12 |
|  | C17-030 |  |  | FJ | Fuan, Ningde, Fujian, China | h15 |  |  | ○ |  |  |  |  | 2017.08.12 |
|  | C17-031 |  |  | FJ | Fuan, Ningde, Fujian, China | h15 |  |  | ○ |  |  |  |  | 2017.08.12 |
|  | C17-032 | C17-032A | *B. nipponica* | FJ | Fuan, Ningde, Fujian, China | h15 |  | ○ | ○ | ○ |  | ○ | ○ | 2017.08.12 |
|  |  | C17-032B | *B. nipponica* | FJ |  |  |  |  |  | ○ |  | ○ | ○ |  |
|  | C17-033 |  |  | FJ | Jiaocheng, Ningde, Fujian, China | h15 |  |  | ○ |  |  |  |  | 2017.08.13 |
|  | C17-035 |  |  | FJ | Jiaocheng, Ningde, Fujian, China | h15 |  |  | ○ |  |  |  |  | 2017.08.13 |
|  | C17-036 |  |  | FJ | Jiaocheng, Ningde, Fujian, China | h15 |  |  | ○ |  |  |  |  | 2017.08.13 |
|  | C17-037 | C17-037A | *B. nipponica* | FJ | Jiaocheng, Ningde, Fujian, China | h14 |  |  | ○ | ○ |  | ○ | ○ | 2017.08.13 |
|  |  | C17-037B | *B. nipponica* | FJ |  |  |  |  |  | ○ |  | ○ | ○ |  |
|  |  | C17-037C | *B. nipponica* | FJ |  |  |  |  |  | ○ |  | ○ | ○ |  |
|  |  | C17-037D | *B. nipponica* | FJ |  |  |  |  |  | ○ |  | ○ | ○ |  |
|  |  | C17-037E | *B. nipponica* | FJ |  |  |  |  |  | ○ |  | ○ | ○ |  |
|  | C17-038 | C17-038A | *B. nipponica* | FJ | Jiaocheng, Ningde, Fujian, China | h15 |  |  | ○ | ○ |  | ○ | ○ | 2017.08.13 |
|  | C17-039 | C17-039A | *B. silvestriana* | FJ | Jiaocheng, Ningde, Fujian, China | h15 |  |  | ○ | ○ | ○ | ○ |  | 2017.08.13 |
|  | C17-050 |  |  | FJ | Jiaocheng, Ningde, Fujian, China | h15 |  |  | ○ |  |  |  |  | 2017.08.13 |
|  | C17-051 |  |  | FJ | Jiaocheng, Ningde, Fujian, China | h15 |  |  | ○ |  |  |  |  | 2017.08.13 |
| Sub-total | 31 | 24 |  | FJ |  | 31 |  | 5 | 31 | 24 | 6 | 24 | 22 |  |
|  | C17-057 |  |  | GD | Mt. Yangtian, Xingyu, Guangdong, China | h18 |  |  | ○ |  |  |  |  | 2017.08.15 |
|  | C17-058 | C17-58mC | *B. silvestriana* | GD | Mt. Yangtian, Xingyu, Guangdong, China | h18 |  |  | ○ | ○ |  | ○ |  | 2017.08.15 |
|  | C17-059 |  |  | GD | Mt. Yangtian, Xingyu, Guangdong, China | h18 |  |  | ○ |  |  |  |  | 2017.08.15 |
|  | C17-060 |  |  | GD | Mt. Yangtian, Xingyu, Guangdong, China |  |  |  | ○ |  |  |  |  | 2017.08.15 |
|  | C17-062 |  |  | GD | Mt. Yangtian, Xingyu, Guangdong, China | h18 |  |  | ○ |  |  |  |  | 2017.08.15 |
|  | C17-063 |  |  | GD | Mt. Yangtian, Xingyu, Guangdong, China | h18 |  |  | ○ |  |  |  |  | 2017.08.15 |
|  | C17-064 | C17-064A | *B. silvestriana* | GD | Mt. Yangtian, Xingyu, Guangdong, China | h18 |  |  | ○ | ○ |  | ○ |  | 2017.08.15 |
|  |  | C17-064B | *B. silvestriana* | GD |  |  |  |  |  | ○ |  | ○ |  |  |
|  |  | C17-064C | *B. silvestriana* | GD |  |  |  |  |  | ○ |  | ○ |  |  |
|  |  | C17-064D | *B. silvestriana* | GD |  |  |  |  |  | ○ |  | ○ |  |  |
|  |  | C17-064E | *B. silvestriana* | GD |  |  |  |  |  | ○ |  | ○ |  |  |
|  | C17-065 |  |  | GD | Mt. Yangtian, Xingyu, Guangdong, China |  |  |  | ○ |  |  |  |  | 2017.08.15 |
|  | C17-066 |  |  | GD | Mt. Yangtian, Xingyu, Guangdong, China | h18 |  |  | ○ |  |  |  |  | 2017.08.15 |
|  | C17-067 |  |  | GD | Mt. Yangtian, Xingyu, Guangdong, China | h18 |  |  | ○ |  |  |  |  | 2017.08.15 |
|  | C17-073 |  |  | GD | Conghua, Guangzhou, Guangdong, China | h19 |  | ○ | ○ |  |  |  |  | 2017.08.17 |
|  | C17-087 |  |  | GD | Conghua, Guangzhou, Guangdong, China | h19 |  | ○ | ○ |  |  |  |  | 2017.08.17 |
|  | C17-088 |  |  | GD | Conghua, Guangzhou, Guangdong, China | h19 |  |  | ○ |  |  |  |  | 2017.08.17 |
|  | C17-089 |  |  | GD | Conghua, Guangzhou, Guangdong, China | h19 |  |  | ○ |  |  |  |  | 2017.08.17 |
|  | C17-090 |  |  | GD | Conghua, Guangzhou, Guangdong, China | h19 |  |  | ○ |  |  |  |  | 2017.08.17 |
|  | C17-091 |  |  | GD | Conghua, Guangzhou, Guangdong, China | h19 |  |  | ○ |  |  |  |  | 2017.08.17 |
|  | C17-092 |  |  | GD | Conghua, Guangzhou, Guangdong, China | h19 |  |  | ○ |  |  |  |  | 2017.08.17 |
|  | C17-093 |  |  | GD | Conghua, Guangzhou, Guangdong, China | h19 |  |  | ○ |  |  |  |  | 2017.08.17 |
|  | C17-094 |  |  | GD | Conghua, Guangzhou, Guangdong, China | h19 |  |  | ○ |  |  |  |  | 2017.08.17 |
|  | C17-095 |  |  | GD | Conghua, Guangzhou, Guangdong, China | h19 |  |  | ○ |  |  |  |  | 2017.08.17 |
|  | C17-096 |  |  | GD | Conghua, Guangzhou, Guangdong, China | h19 |  |  | ○ |  |  |  |  | 2017.08.17 |
|  | C17-105 |  |  | GD | Conghua, Guangzhou, Guangdong, China | h19 |  |  | ○ |  |  |  |  | 2017.08.17 |
|  | C17-139 |  |  | GD | Conghua, Guangzhou, Guangdong, China | h19 |  |  | ○ |  |  |  |  | 2017.08.18 |
|  | C17-140 |  |  | GD | Conghua, Guangzhou, Guangdong, China | h19 |  |  | ○ |  |  |  |  | 2017.08.18 |
|  | C17-141 |  |  | GD | Conghua, Guangzhou, Guangdong, China | h19 |  |  | ○ |  |  |  |  | 2017.08.18 |
|  | C17-142 |  |  | GD | Conghua, Guangzhou, Guangdong, China | h19 |  |  | ○ |  |  |  |  | 2017.08.18 |
|  | C17-143 |  |  | GD | Conghua, Guangzhou, Guangdong, China | h19 |  |  | ○ |  |  |  |  | 2017.08.18 |
|  | C18-004 |  |  | GD | Conghua, Guangzhou, Guangdong, China | h15 |  |  | ○ |  |  |  |  | 2018.04.24 |
|  | C18-005 |  |  | GD | Conghua, Guangzhou, Guangdong, China | h15 |  |  | ○ |  |  |  |  | 2018.04.24 |
|  | C18-011 |  |  | GD | Conghua, Guangzhou, Guangdong, China | h19 |  | ○ | ○ |  |  |  |  | 2018.04.24 |
|  | C18-012 |  |  | GD | Conghua, Guangzhou, Guangdong, China | h19 |  |  | ○ |  |  |  |  | 2018.04.24 |
|  | C18-013 |  |  | GD | Conghua, Guangzhou, Guangdong, China |  |  |  | ○ |  |  |  |  | 2018.04.24 |
|  | C18-014 |  |  | GD | Conghua, Guangzhou, Guangdong, China | h19 |  | ○ | ○ |  |  |  |  | 2018.04.24 |
|  | C18-015 |  |  | GD | Conghua, Guangzhou, Guangdong, China | h15 |  |  | ○ |  |  |  |  | 2018.04.24 |
|  | C18-016 |  |  | GD | Conghua, Guangzhou, Guangdong, China | h19 |  | ○ | ○ |  |  |  |  | 2018.04.24 |
|  | C18-017 |  |  | GD | Conghua, Guangzhou, Guangdong, China | h15 |  |  | ○ |  |  |  |  | 2018.04.24 |
|  | C18-018 |  |  | GD | Conghua, Guangzhou, Guangdong, China | h15 |  |  | ○ |  |  |  |  | 2018.04.24 |
|  | C18-032 |  |  | GD | Conghua, Guangzhou, Guangdong, China |  |  |  | ○ |  |  |  |  | 2018.04.24 |
|  | C18-093 | C18-093A | *B. silvestriana* | GD | Conghua, Guangzhou, Guangdong, China |  |  |  |  | ○ | ○ | ○ |  | 2018.06.23 |
|  |  | C18-093B | *B. silvestriana* | GD |  |  |  |  |  |  | ○ | ○ |  |  |
|  |  | C18-093C | *B. silvestriana* | GD |  |  |  |  |  |  |  | ○ |  |  |
|  |  | C18-093D | *B. silvestriana* | GD |  |  |  |  |  |  |  | ○ |  |  |
|  |  | C18-093E | *B. silvestriana* | GD |  |  |  |  |  |  |  | ○ |  |  |
|  | C18-094 | C18-094A | *B. silvestriana* | GD | Conghua, Guangzhou, Guangdong, China |  |  |  |  |  |  | ○ |  | 2018.06.23 |
|  |  | C18-094B | *B. silvestriana* | GD |  |  |  |  |  |  |  | ○ |  |  |
|  |  | C18-094C | *B. silvestriana* | GD |  |  |  |  |  |  |  | ○ |  |  |
|  |  | C18-094D | *B. silvestriana* | GD |  |  |  |  |  |  |  | ○ |  |  |
|  |  | C18-094E | *B. silvestriana* | GD |  |  |  |  |  |  |  | ○ |  |  |
|  | C18-095 | C18-095A | *B. silvestriana* | GD | Conghua, Guangzhou, Guangdong, China |  |  |  |  |  |  | ○ |  | 2018.06.23 |
|  |  | C18-095B | *B. silvestriana* | GD |  |  |  |  |  |  |  | ○ |  |  |
|  |  | C18-095C | *B. silvestriana* | GD |  |  |  |  |  |  |  | ○ |  |  |
|  |  | C18-095D | *B. silvestriana* | GD |  |  |  |  |  |  |  | ○ |  |  |
|  |  | C18-095E | *B. silvestriana* | GD |  |  |  |  |  |  |  | ○ |  |  |
|  | C18-096 | C18-096A | *B. silvestriana* | GD | Conghua, Guangzhou, Guangdong, China |  |  |  |  | ○ |  | ○ |  | 2018.06.23 |
|  |  | C18-096B | *B. silvestriana* | GD |  |  |  |  |  |  |  | ○ |  |  |
|  |  | C18-096C | *B. silvestriana* | GD |  |  |  |  |  |  |  | ○ |  |  |
|  |  | C18-096D | *B. silvestriana* | GD |  |  |  |  |  |  |  | ○ |  |  |
|  |  | C18-096E | *B. silvestriana* | GD |  |  |  |  |  |  |  | ○ |  |  |
| Sub-total | 42 | 26 |  | GD |  | 34 |  | 5 | 38 | 8 | 2 | 26 |  |  |
|  | C18-054 |  |  | HK | Mt. Taimo-san, Hong Kong | h14 |  |  | ○ |  |  |  |  | 2018.04.28 |
|  | C18-055 |  |  | HK | Mt. Taimo-san, Hong Kong | h14 | ○ |  | ○ |  |  |  |  | 2018.04.28 |
|  | C18-056 |  |  | HK | Mt. Taimo-san, Hong Kong | h14 | ○ |  | ○ |  |  |  |  | 2018.04.28 |
|  | C18-057 |  |  | HK | Mt. Taimo-san, Hong Kong |  |  |  | ○ |  |  |  |  | 2018.04.28 |
|  | C18-058 |  |  | HK | Mt. Taimo-san, Hong Kong | h14 | ○ |  | ○ |  |  |  |  | 2018.04.28 |
|  | C18-059 |  |  | HK | Mt. Taimo-san, Hong Kong | h14 | ○ |  | ○ |  |  |  |  | 2018.04.28 |
|  | C18-060 |  |  | HK | Mt. Taimo-san, Hong Kong | h14 | ○ |  | ○ |  |  |  |  | 2018.04.28 |
|  | C18-061 |  |  | HK | Mt. Taimo-san, Hong Kong | h14 |  |  | ○ |  |  |  |  | 2018.04.28 |
|  | C18-062 |  |  | HK | Mt. Taimo-san, Hong Kong | h14 |  |  | ○ |  |  |  |  | 2018.04.28 |
|  | C18-067 |  |  | HK | Pat Sin Leng Country Park, Hong Kong |  |  |  | ○ |  |  |  |  | 2018.04.28 |
|  | C18-073 | C18-073mA | *B. silvestriana* | HK | Sai kung East Country Park, Hong Kong | h7 |  |  |  |  |  | ○ |  | 2018.04.29 |
|  |  | C18-073mB | *B. silvestriana* | HK |  |  |  |  |  | ○ | ○ | ○ |  |  |
|  |  | C18-073mC | *B. silvestriana* | HK |  |  |  |  |  | ○ | ○ | ○ |  |  |
|  | C18-076 |  |  | HK | Sai kung East Country Park, Hong Kong | h7 |  |  |  |  |  |  |  | 2018.04.29 |
|  | C18-077 |  |  | HK | Sai kung East Country Park, Hong Kong | h7 |  |  |  |  |  |  |  | 2018.04.29 |
|  | C18-081 |  |  | HK | Sai kung East Country Park, Hong Kong | h7 |  |  |  |  |  |  |  | 2018.04.29 |
| Sub-total | 14 | 3 |  | HK |  | 12 | 5 |  | 10 | 2 | 2 | 3 |  |  |
| *F. formosana* | C17-074 |  |  | GD | Conghua, Guangzhou, Guangdong, China | h20 |  |  | ○ |  |  |  |  | 2017.08.17 |
|  | C17-075 |  |  | GD | Conghua, Guangzhou, Guangdong, China | h21 |  |  | ○ |  |  |  |  | 2017.08.17 |
|  | C17-076 |  |  | GD | Conghua, Guangzhou, Guangdong, China | h21 |  |  | ○ |  |  |  |  | 2017.08.17 |
|  | C17-077 |  |  | GD | Conghua, Guangzhou, Guangdong, China | h21 |  |  | ○ |  |  |  |  | 2017.08.17 |
|  | C17-080 |  |  | GD | Conghua, Guangzhou, Guangdong, China | h20 |  |  | ○ |  |  |  |  | 2017.08.17 |
|  | C17-081 |  |  | GD | Conghua, Guangzhou, Guangdong, China | h20 |  |  | ○ |  |  |  |  | 2017.08.17 |
|  | C17-086 |  |  | GD | Conghua, Guangzhou, Guangdong, China | h21 |  |  | ○ |  |  |  |  | 2017.08.17 |
|  | C17-116 | C17-116B | *B. silvestriana* | GD | Conghua, Guangzhou, Guangdong, China | h21 |  |  |  | ○ |  | ○ |  | 2017.08.17 |
|  |  | C17-116C | *B. silvestriana* | GD |  |  |  |  |  | ○ |  | ○ |  |  |
|  | C17-126 |  |  | GD | Conghua, Guangzhou, Guangdong, China | h21 |  |  | ○ |  |  |  |  | 2017.08.17 |
|  | C17-127 |  |  | GD | Conghua, Guangzhou, Guangdong, China | h21 |  |  | ○ |  |  |  |  | 2017.08.17 |
|  | C17-128 |  |  | GD | Conghua, Guangzhou, Guangdong, China | h21 |  |  | ○ |  |  |  |  | 2017.08.17 |
|  | C17-129 |  |  | GD | Conghua, Guangzhou, Guangdong, China | h21 |  |  | ○ |  |  |  |  | 2017.08.17 |
|  | C17-130 |  |  | GD | Conghua, Guangzhou, Guangdong, China | h21 |  |  | ○ |  |  |  |  | 2017.08.17 |
|  | C17-131 |  |  | GD | Conghua, Guangzhou, Guangdong, China | h21 |  |  | ○ |  |  |  |  | 2017.08.17 |
|  | C17-133 |  |  | GD | Conghua, Guangzhou, Guangdong, China | h21 |  |  | ○ |  |  |  |  | 2017.08.17 |
|  | C17-134 |  |  | GD | Conghua, Guangzhou, Guangdong, China | h21 |  |  | ○ |  |  |  |  | 2017.08.17 |
|  | C17-135 |  |  | GD | Conghua, Guangzhou, Guangdong, China | h21 |  |  | ○ |  |  |  |  | 2017.08.17 |
|  | C17-137 |  |  | GD | Conghua, Guangzhou, Guangdong, China | h21 |  |  | ○ |  |  |  |  | 2017.08.17 |
|  | C18-001 | C18-001A | *B. silvestriana* | GD | Conghua, Guangzhou, Guangdong, China | h21 |  | ○ | ○ |  |  | ○ |  | 2018.04.24 |
|  |  | C18-001B | *B. silvestriana* | GD |  |  |  |  |  |  |  | ○ |  |  |
|  |  | C18-001C | *B. silvestriana* | GD |  |  |  |  |  |  |  | ○ |  |  |
|  |  | C18-001D | *B. silvestriana* | GD |  |  |  |  |  |  |  | ○ |  |  |
|  |  | C18-001E | *B. silvestriana* | GD |  |  |  |  |  |  |  | ○ |  |  |
|  | C18-002 |  |  | GD | Conghua, Guangzhou, Guangdong, China | h21 |  |  | ○ |  |  |  |  | 2018.04.24 |
|  | C18-003 |  |  | GD | Conghua, Guangzhou, Guangdong, China | h21 |  |  | ○ |  |  |  |  | 2018.04.24 |
|  | C18-006 |  |  | GD | Conghua, Guangzhou, Guangdong, China | h21 |  |  | ○ |  |  |  |  | 2018.04.24 |
|  | C18-007 |  |  | GD | Conghua, Guangzhou, Guangdong, China | h21 |  |  | ○ |  |  |  |  | 2018.04.24 |
|  | C18-008 |  |  | GD | Conghua, Guangzhou, Guangdong, China | h21 |  |  | ○ |  |  |  |  | 2018.04.24 |
|  | C18-009 |  |  | GD | Conghua, Guangzhou, Guangdong, China | h21 |  |  | ○ |  |  |  |  | 2018.04.24 |
|  | C18-010 | C18-010A | *B. silvestriana* | GD | Conghua, Guangzhou, Guangdong, China | h21 |  | ○ | ○ |  | ○ | ○ |  | 2018.04.24 |
|  |  | C18-010B | *B. silvestriana* | GD |  |  |  |  |  |  | ○ | ○ |  |  |
|  | C18-020 | C18-020A | *B. silvestriana* | GD | Conghua, Guangzhou, Guangdong, China | h20 |  |  | ○ |  |  | ○ |  | 2018.04.24 |
|  |  | C18-020B | *B. silvestriana* | GD |  |  |  |  |  |  |  | ○ |  |  |
|  |  | C18-020C | *B. silvestriana* | GD |  |  |  |  |  |  |  | ○ |  |  |
|  | C18-021 |  |  | GD | Conghua, Guangzhou, Guangdong, China | h21 |  |  | ○ |  |  |  |  | 2018.04.24 |
|  | C18-022 | C18-022 | *B. silvestriana* | GD | Conghua, Guangzhou, Guangdong, China | h20 |  | ○ | ○ |  |  | ○ |  | 2018.04.24 |
|  | C18-023 | C18-023A | *B. silvestriana* | GD | Conghua, Guangzhou, Guangdong, China | h20 |  |  | ○ |  |  | ○ |  | 2018.04.24 |
|  |  | C18-023B | *B. silvestriana* | GD |  |  |  |  |  |  |  | ○ |  |  |
|  | C18-024 | C18-024A | *B. silvestriana* | GD | Conghua, Guangzhou, Guangdong, China | h20 |  |  | ○ |  |  | ○ |  | 2018.04.24 |
|  |  | C18-024B | *B. silvestriana* | GD |  |  |  |  |  |  |  | ○ |  |  |
|  | C18-025 | C18-025A | *B. silvestriana* | GD | Conghua, Guangzhou, Guangdong, China | h20 |  |  | ○ |  |  | ○ |  | 2018.04.24 |
|  |  | C18-025B | *B. silvestriana* | GD |  |  |  |  |  |  |  | ○ |  |  |
|  | C18-028 |  |  | GD | Conghua, Guangzhou, Guangdong, China | h21 |  |  | ○ |  |  |  |  | 2018.04.24 |
|  | C18-029 | C18-029 | *B. silvestriana* | GD | Conghua, Guangzhou, Guangdong, China | h21 |  | ○ | ○ |  |  | ○ |  | 2018.04.24 |
|  | C18-030 | C18-030A | *B. silvestriana* | GD | Conghua, Guangzhou, Guangdong, China | h21 |  | ○ | ○ |  |  | ○ |  | 2018.04.24 |
|  |  | C18-030B | *B. silvestriana* | GD |  |  |  |  |  |  |  | ○ |  |  |
| Sub-total | 35 | 22 |  | GD |  | 35 |  | 5 | 34 | 2 | 2 | 22 |  |  |
|  | C17-168 | C17-168m | *B. silvestriana* | HK | Mt. Kowloon, Hong Kong | h9 |  |  | ○ | ○ |  | ○ |  | 2017.08.20 |
|  | C17-169 |  |  | HK | Mt. Kowloon, Hong Kong | h9 |  |  | ○ |  |  |  |  | 2017.08.20 |
|  | C17-170 |  |  | HK | Mt. Kowloon, Hong Kong | h9 |  |  | ○ |  |  |  |  | 2017.08.20 |
|  | C17-171 |  |  | HK | Mt. Kowloon, Hong Kong | h9 |  |  | ○ |  |  |  |  | 2017.08.20 |
|  | C17-172 |  |  | HK | Mt. Kowloon, Hong Kong | h9 |  |  | ○ |  |  |  |  | 2017.08.20 |
|  | C17-173 |  |  | HK | Mt. Kowloon, Hong Kong | h9 |  |  | ○ |  |  |  |  | 2017.08.20 |
|  | C17-174 |  |  | HK | Mt. Kowloon, Hong Kong | h9 |  |  | ○ |  |  |  |  | 2017.08.20 |
|  | C17-175 |  |  | HK | Mt. Kowloon, Hong Kong | h9 |  |  | ○ |  |  |  |  | 2017.08.20 |
|  | C17-176 | C17-176A | *B. silvestriana* | HK | Mt. Kowloon, Hong Kong | h9 |  |  | ○ |  |  | ○ |  | 2017.08.20 |
|  |  | C17-176B | *B. silvestriana* | HK |  |  |  |  |  |  |  | ○ |  |  |
|  |  | C17-176C | *B. silvestriana* | HK |  |  |  |  |  |  |  | ○ |  |  |
|  |  | C17-176D | *B. silvestriana* | HK |  |  |  |  |  |  |  | ○ |  |  |
|  |  | C17-176E | *B. silvestriana* | HK |  |  |  |  |  |  |  | ○ |  |  |
|  | C17-177 |  |  | HK | Mt. Kowloon, Hong Kong | h9 |  |  | ○ |  |  |  |  | 2017.08.20 |
|  | C17-178 |  |  | HK | Mt. Kowloon, Hong Kong | h9 |  |  | ○ |  |  |  |  | 2017.08.20 |
|  | C17-179 |  |  | HK | Mt. Kowloon, Hong Kong | h9 |  |  | ○ |  |  |  |  | 2017.08.20 |
|  | C17-180 |  |  | HK | Mt. Kowloon, Hong Kong | h9 |  |  | ○ |  |  |  |  | 2017.08.20 |
|  | C17-181 |  |  | HK | Mt. Kowloon, Hong Kong | h9 |  |  | ○ |  |  |  |  | 2017.08.20 |
|  | C17-182 |  |  | HK | Mt. Kowloon, Hong Kong | h9 |  |  | ○ |  |  |  |  | 2017.08.20 |
|  | C17-183 | C17-183A | *B. silvestriana* | HK | Mt. Kowloon, Hong Kong | h9 |  |  | ○ |  |  | ○ |  | 2017.08.20 |
|  |  | C17-183B | *B. silvestriana* | HK |  |  |  |  |  |  |  | ○ |  |  |
|  |  | C17-183C | *B. silvestriana* | HK |  |  |  |  |  |  |  | ○ |  |  |
|  |  | C17-183D | *B. silvestriana* | HK |  |  |  |  |  |  |  | ○ |  |  |
|  |  | C17-183E | *B. silvestriana* | HK |  |  |  |  |  |  |  | ○ |  |  |
|  | C17-184 |  |  | HK | Mt. Kowloon, Hong Kong | h9 |  |  | ○ |  |  |  |  | 2017.08.20 |
|  | C17-188 |  |  | HK | Mt. Kowloon, Hong Kong | h9 |  |  | ○ |  |  |  |  | 2017.08.20 |
|  | C17-189 |  |  | HK | Mt. Kowloon, Hong Kong | h9 |  |  | ○ |  |  |  |  | 2017.08.20 |
|  | C17-193 |  |  | HK | Lions Nature Education Centre, Hong Kong | h9 |  |  | ○ |  |  |  |  | 2017.08.20 |
|  | C17-194 |  |  | HK | Lions Nature Education Centre, Hong Kong | h9 |  |  | ○ |  |  |  |  | 2017.08.20 |
|  | C17-195 |  |  | HK | Lions Nature Education Centre, Hong Kong | h9 |  |  | ○ |  |  |  |  | 2017.08.20 |
|  | C17-196 |  |  | HK | Lions Nature Education Centre, Hong Kong | h9 |  |  | ○ |  |  |  |  | 2017.08.20 |
|  | C17-197 |  |  | HK | Lions Nature Education Centre, Hong Kong | h9 |  |  | ○ |  |  |  |  | 2017.08.20 |
|  | C18-066 |  |  | HK | Pat Sin Leng Country Park, Hong Kong | h9 |  |  | ○ |  |  |  |  | 2018.04.28 |
|  | C18-068 |  |  | HK | Pat Sin Leng Country Park, Hong Kong | h9 |  |  | ○ |  |  |  |  | 2018.04.28 |
|  | C18-071 |  |  | HK | Sai kung East Country Park, Hong Kong | h9 |  |  | ○ |  |  |  |  | 2018.04.29 |
|  | C18-072 |  |  | HK | Sai kung East Country Park, Hong Kong | h9 | ○ |  | ○ |  |  |  |  | 2018.04.29 |
|  | C18-074 | C18-074mA | *B. silvestriana* | HK | Sai kung East Country Park, Hong Kong | h9 | ○○* |  | ○ |  |  | ○ |  | 2018.04.29 |
|  | C18-075 |  |  | HK | Sai kung East Country Park, Hong Kong | h9 | ○ |  | ○ |  |  |  |  | 2018.04.29 |
|  | C18-080 |  |  | HK | Sai kung East Country Park, Hong Kong | h9 |  |  | ○ |  |  |  |  | 2018.04.29 |
|  | C18-082 |  |  | HK | Sai kung East Country Park, Hong Kong | h9 |  |  | ○ |  |  |  |  | 2018.04.29 |
|  | C18-085 | C18-085A | *B. silvestriana* | HK | Mt. Kowloon, Hong Kong | h9 | ○ |  | ○ |  | ○ | ○ |  | 2018.04.30 |
|  |  | C18-085B | *B. silvestriana* | HK |  |  |  |  |  | ○ | ○ | ○ |  |  |
|  |  | C18-085C | *B. silvestriana* | HK |  |  |  |  |  |  |  | ○ |  |  |
|  |  | C18-085D | *B. silvestriana* | HK |  |  |  |  |  |  |  | ○ |  |  |
|  |  | C18-085E | *B. silvestriana* | HK |  |  |  |  |  |  |  | ○ |  |  |
|  | C18-086 | C18-086 | *B. silvestriana* | HK | Mt. Kowloon, Hong Kong | h9 | ○ |  | ○ |  |  | ○ |  | 2018.04.30 |
|  | C18-088 | C18-088mA | *B. silvestriana* | HK | Mt. Kowloon, Hong Kong | h9 | ○○* |  | ○ |  |  | ○ |  | 2018.04.30 |
| Sub-total | 35 | 19 |  | HK |  | 35 | 8 |  | 35 | 2 | 2 | 19 |  |  |
|  | TW11-017 |  |  | TW | Lienhuachih Research Center, Nantou, Taiwan | h9 |  |  | ● |  |  |  |  | 2011.09.06 |
|  | TW11-018 |  |  | TW | Lienhuachih Research Center, Nantou, Taiwan | h9 |  |  | ● |  |  |  |  | 2011.09.06 |
|  | TW11-048 | TW11-048-1 | *B. taiwanensis* | TW | Huisun Forest Area, Nantou, Taiwan | h9 |  |  | ● |  |  | ● | ● | 2011.09.07 |
|  | TW11-049 |  |  | TW | Huisun Forest Area, Nantou, Taiwan | h9 |  |  | ● |  |  |  |  | 2011.09.07 |
|  | TW11-050 |  |  | TW | Huisun Forest Area, Nantou, Taiwan | h9 |  |  | ● |  |  |  |  | 2011.09.07 |
|  | TW11-051 |  |  | TW | Huisun Forest Area, Nantou, Taiwan | h9 |  |  | ● |  |  |  |  | 2011.09.07 |
|  | TW11-052 |  |  | TW | Huisun Forest Area, Nantou, Taiwan | h9 |  |  | ● |  |  |  |  | 2011.09.07 |
|  | TW11-053 |  |  | TW | Huisun Forest Area, Nantou, Taiwan | h9 |  |  | ● |  |  |  |  | 2011.09.07 |
|  | TW11-054 |  |  | TW | Huisun Forest Area, Nantou, Taiwan | h9 |  |  | ● |  |  |  |  | 2011.09.07 |
|  | TW11-078 |  |  | TW | Manzhou, Pingdong, Taiwan | h22 |  |  | ● |  |  |  |  | 2011.09.09 |
|  | TW11-079 |  |  | TW | Manzhou, Pingdong, Taiwan | h22 |  |  | ● |  |  |  |  | 2011.09.09 |
|  | TW11-080 |  |  | TW | Manzhou, Pingdong, Taiwan | h22 |  |  | ● |  |  |  |  | 2011.09.09 |
|  | TW11-081 |  |  | TW | Manzhou, Pingdong, Taiwan | h22 |  |  | ● |  |  |  |  | 2011.09.09 |
|  | TW11-082 |  |  | TW | Manzhou, Pingdong, Taiwan | h22 |  |  | ● |  |  |  |  | 2011.09.09 |
|  | TW11-083 |  |  | TW | Manzhou, Pingdong, Taiwan | h22 |  |  | ● |  |  |  |  | 2011.09.09 |
|  | TW11-084 |  |  | TW | Manzhou, Pingdong, Taiwan | h22 |  |  | ● |  |  |  |  | 2011.09.09 |
|  | TW11-085 |  |  | TW | Manzhou, Pingdong, Taiwan | h22 |  |  | ● |  |  |  |  | 2011.09.09 |
|  | TW11-086 |  |  | TW | Manzhou, Pingdong, Taiwan | h22 |  |  | ● |  |  |  |  | 2011.09.09 |
|  | TW11-087 |  |  | TW | Manzhou, Pingdong, Taiwan | h22 |  |  | ● |  |  |  |  | 2011.09.09 |
|  | TW11-088 |  |  | TW | Manzhou, Pingdong, Taiwan | h9 |  |  | ● |  |  |  |  | 2011.09.09 |
|  | TW11-089 |  |  | TW | Manzhou, Pingdong, Taiwan | h9 |  |  | ● |  |  |  |  | 2011.09.09 |
|  | TW13-002 | TW13-002a | *B. taiwanensis* | TW | Jufu, Changbin, Taidong, Taiwan | h22 |  | ○ | ○ |  |  |  | ● | 2013.03.19 |
|  |  | TW13-002b | *B. taiwanensis* | TW |  |  |  |  |  |  |  | ● | ● |  |
|  |  | TW13-002c | *B. taiwanensis* | TW |  |  |  |  |  |  |  | ● | ● |  |
|  |  | TW13-002d | *B. taiwanensis* | TW |  |  |  |  |  |  |  | ● | ● |  |
|  | TW13-003 | TW13-003a | *B. taiwanensis* | TW | Jufu, Changbin, Taidong, Taiwan | h22 |  | ○ | ○ |  |  |  | ● | 2013.03.19 |
|  |  | TW13-003b | *B. taiwanensis* | TW |  |  |  |  |  |  |  |  | ● |  |
|  | TW13-004 |  |  | TW | Jufu, Changbin, Taidong, Taiwan | h22 |  |  | ○ |  |  |  |  | 2013.03.19 |
|  | TW13-005 | TW13-005 | *B. taiwanensis* | TW | Jufu, Changbin, Taidong, Taiwan | h22 |  | ○ | ○ |  |  | ● | ● | 2013.03.19 |
|  | TW13-009 |  |  | TW | Changbin, Taidong, Taiwan | h22 |  | ○ | ○ |  |  |  |  | 2013.03.19 |
|  | TW13-011 |  |  | TW | Changbin, Taidong, Taiwan | h22 |  | ○ | ○ |  |  |  |  | 2013.03.19 |
|  | TW13-012 |  |  | TW | Changbin, Taidong, Taiwan | h22 |  |  | ○ |  |  |  |  | 2013.03.19 |
|  | TW13-013 |  |  | TW | Changbin, Taidong, Taiwan | h22 |  |  | ○ |  |  |  |  | 2013.03.19 |
|  | TW13-015 |  |  | TW | Changbin, Taidong, Taiwan | h22 |  |  | ○ |  |  |  |  | 2013.03.19 |
|  | TW13-016 |  |  | TW | Changbin, Taidong, Taiwan | h22 |  |  | ○ |  |  |  |  | 2013.03.19 |
|  | TW13-017 |  |  | TW | Changbin, Taidong, Taiwan | h22 |  |  | ○ |  |  |  |  | 2013.03.19 |
|  | TW13-021 |  |  | TW | Changbin, Taidong, Taiwan | h22 |  |  | ○ |  |  |  |  | 2013.03.19 |
|  | TW13-083 |  |  | TW | Kenting, Pingdong, Taiwan | h9 |  |  | ○ |  |  |  |  | 2013.03.24 |
|  | TW13-084 |  |  | TW | Kenting, Pingdong, Taiwan | h9 |  |  | ○ |  |  |  |  | 2013.03.24 |
|  | TW13-085 |  |  | TW | Kenting, Pingdong, Taiwan | h9 |  |  | ○ |  |  |  |  | 2013.03.24 |
|  | TW13-086 |  |  | TW | Kenting, Pingdong, Taiwan | h9 |  |  | ○ |  |  |  |  | 2013.03.24 |
|  | TW13-087 |  |  | TW | Kenting, Pingdong, Taiwan | h9 |  |  | ○ |  |  |  |  | 2013.03.24 |
|  | TW13-088 |  |  | TW | Kenting, Pingdong, Taiwan | h9 |  |  | ○ |  |  |  |  | 2013.03.24 |
|  | TW13-089 |  |  | TW | Kenting, Pingdong, Taiwan | h9 |  |  | ○ |  |  |  |  | 2013.03.24 |
|  | TW14-001 | TW14-001-1a | *B. taiwanensis* | TW | Jufu, Changbin, Taidong, Taiwan |  |  |  |  | ○ |  | ○ | ○ | 2014.03.21 |
|  |  | TW14-001-1b | *B. taiwanensis* | TW |  |  |  |  |  | ○ |  |  | ○ |  |
|  |  | TW14-001-1c | *B. taiwanensis* | TW |  |  |  |  |  | ○ |  |  | ○ |  |
|  |  | TW14-001-1e | *B. taiwanensis* | TW |  |  |  |  |  | ○ |  |  | ○ |  |
|  |  | TW14-001-1f | *B. taiwanensis* | TW |  |  |  |  |  | ○ |  |  | ○ |  |
|  | TW14-002 | TW14-002-a1 | *B. taiwanensis* | TW | Jufu, Changbin, Taidong, Taiwan |  |  |  |  | ○ |  | ○ | ○ | 2014.03.21 |
|  |  | TW14-002-a2 | *B. taiwanensis* | TW |  |  |  |  |  | ○ |  |  | ○ |  |
|  |  | TW14-002-b1 | *B. taiwanensis* | TW |  |  |  |  |  | ○ |  |  | ○ |  |
|  |  | TW14-002-c | *B. taiwanensis* | TW |  |  |  |  |  | ○ |  |  | ○ |  |
|  |  | TW14-002-d | *B. taiwanensis* | TW |  |  |  |  |  | ○ |  |  | ○ |  |
|  | TW14-003 | TW14-003-a | *B. taiwanensis* | TW | Jufu, Changbin, Taidong, Taiwan |  |  |  |  | ○ |  |  | ○ | 2014.03.21 |
|  |  | TW14-003-b | *B. taiwanensis* | TW |  |  |  |  |  | ○ |  |  | ○ |  |
|  |  | TW14-003-c | *B. taiwanensis* | TW |  |  |  |  |  | ○ |  |  | ○ |  |
|  |  | TW14-003-d | *B. taiwanensis* | TW |  |  |  |  |  | ○ |  |  | ○ |  |
|  |  | TW14-003-e | *B. taiwanensis* | TW |  |  |  |  |  | ○ |  |  | ○ |  |
|  | TW16-180 |  |  | TW | Guishandao, Toucheng, Yilan, Taiwan | h9 |  |  | ○ |  |  |  |  | 2016.08.12 |
|  | TW16-181 |  |  | TW | Guishandao, Toucheng, Yilan, Taiwan | h9 |  |  | ○ |  |  |  |  | 2016.08.12 |
|  | TW16-182 |  |  | TW | Guishandao, Toucheng, Yilan, Taiwan | h9 |  |  | ○ |  |  |  |  | 2016.08.12 |
|  | TW16-183 |  |  | TW | Guishandao, Toucheng, Yilan, Taiwan | h9 |  |  | ○ |  |  |  |  | 2016.08.12 |
|  | TW16-185 |  |  | TW | Guishandao, Toucheng, Yilan, Taiwan | h9 |  |  | ○ |  |  |  |  | 2016.08.12 |
|  | TW16-186 |  |  | TW | Guishandao, Toucheng, Yilan, Taiwan | h9 |  |  | ○ |  |  |  |  | 2016.08.12 |
|  | TW16-190 |  |  | TW | Guishandao, Toucheng, Yilan, Taiwan | h9 |  |  | ○ |  |  |  |  | 2016.08.12 |
|  | TW16-194 |  |  | TW | Guishandao, Toucheng, Yilan, Taiwan | h9 |  |  | ○ |  |  |  |  | 2016.08.12 |
|  | TW16-204 |  |  | TW | Guishandao, Toucheng, Yilan, Taiwan | h9 |  |  | ○ |  |  |  |  | 2016.08.12 |
|  | TW16-205 |  |  | TW | Guishandao, Toucheng, Yilan, Taiwan | h9 |  |  | ○ |  |  |  |  | 2016.08.12 |
|  | TW16-221 |  |  | TW | Guishandao, Toucheng, Yilan, Taiwan | h9 |  |  | ○ |  |  |  |  | 2016.08.12 |
|  | TW18-183 | TW18-183 | *B. taiwanensis* | TW | Mudan, Pingtung, Taiwan |  |  |  |  |  |  | ○ | ○ | 2018.08.31 |
|  | TW18-184 | TW18-184 | *B. taiwanensis* | TW | Mudan, Pingtung, Taiwan |  |  |  |  |  |  | ○ | ○ | 2018.08.31 |
|  | TW18-187 | TW18-187A | *B. taiwanensis* | TW | Mudan, Pingtung, Taiwan |  |  |  |  |  | ○ | ○ | ○ | 2018.08.31 |
|  | TW18-232 | TW18-232 | *B. taiwanensis* | TW | Mudan, Pingtung, Taiwan |  |  |  |  |  |  | ○ | ○ | 2018.09.01 |
|  | TW18-233 | TW18-233A | *B. taiwanensis* | TW | Mudan, Pingtung, Taiwan |  |  |  |  |  |  | ○ | ○ | 2018.09.01 |
|  | TW18-241 | TW18-241A | *B. taiwanensis* | TW | Maolin, Gaoxiong, Taiwan |  |  |  |  |  |  | ○ | ○ | 2018.09.02 |
|  | TW18-242 | TW18-242 | *B. taiwanensis* | TW | Maolin, Gaoxiong, Taiwan |  |  |  |  |  | ○ | ○ | ○ | 2018.09.02 |
| Sub-total | 61 | 30 |  | TW |  | 51 |  | 5 | 51 | 15 | 2 | 14 | 30 |  |
| *F. vaccinioides* | TW11-100 | TW11-100 | *B. yeni* | TW | Kenting, Pingdong, Taiwan | h23 |  |  | ● | ○ |  |  | ● | 2011.09.10 |
|  | TW11-184 |  |  | TW | Taroko National Park, Taiwan | h24 |  |  | ● |  |  |  |  | 2011.09.15 |
|  | TW11-194 |  |  | TW | Taroko National Park, Taiwan | h24 |  |  | ● |  |  |  |  | 2011.09.15 |
|  | TW13-001 | TW13-001a | *B. yeni* | TW | Kenting, Pingdong, Taiwan |  |  |  |  |  |  |  | ● | 2013.03.19 |
|  |  | TW13-001b | *B. yeni* | TW |  |  |  |  |  |  |  |  | ● |  |
|  |  | TW13-001c | *B. yeni* | TW |  |  |  |  |  |  |  |  | ● |  |
|  |  | TW13-001d | *B. yeni* | TW |  |  |  |  |  |  |  |  | ● |  |
|  | TW13-022 |  |  | TW | Fengbin, Hualian, Taiwan | h24 |  | ○ | ● |  |  |  |  | 2013.03.19 |
|  | TW13-023 |  |  | TW | Fengbin, Hualian, Taiwan | h24 |  | ○ | ● |  |  |  |  | 2013.03.19 |
|  | TW13-024 |  |  | TW | Fengbin, Hualian, Taiwan | h23 |  |  | ● |  |  |  |  | 2013.03.19 |
|  | TW13-025 |  |  | TW | Fengbin, Hualian, Taiwan | h23 |  |  | ● |  |  |  |  | 2013.03.19 |
|  | TW13-026 |  |  | TW | Fengbin, Hualian, Taiwan | h23 |  |  | ● |  |  |  |  | 2013.03.19 |
|  | TW13-027 |  |  | TW | Fengbin, Hualian, Taiwan | h23 |  |  | ● |  |  |  |  | 2013.03.19 |
|  | TW13-028 |  |  | TW | Fengbin, Hualian, Taiwan | h23 |  |  | ● |  |  |  |  | 2013.03.19 |
|  | TW13-029 | TW13-029a | *B.* sp. | TW | Fengbin, Hualian, Taiwan | h23 |  | ○ | ● |  |  |  | ● | 2013.03.19 |
|  |  | TW13-029b | *B.* sp. | TW |  |  |  |  |  |  |  |  | ● |  |
|  | TW13-030 | TW13-030a | *B.* sp. | TW | Fengbin, Hualian, Taiwan | h23 |  | ○ | ● |  |  |  | ● | 2013.03.19 |
|  |  | TW13-030b | *B.* sp. | TW |  |  |  |  |  |  |  | ● | ● |  |
|  |  | TW13-030c | *B.* sp. | TW |  |  |  |  |  |  |  | ● | ● |  |
|  |  | TW13-030d | *B.* sp. | TW |  |  |  |  |  |  |  |  | ● |  |
|  |  | TW13-030e | *B.* sp. | TW |  |  |  |  |  |  |  | ● | ● |  |
|  | TW13-031 | TW13-031a | *B.* sp. | TW | Fengbin, Hualian, Taiwan | h23 |  | ○ | ● |  |  | ● | ● | 2013.03.19 |
|  |  | TW13-031b | *B.* sp. | TW |  |  |  |  |  |  |  |  | ● |  |
|  |  | TW13-031d | *B.* sp. | TW |  |  |  |  |  |  |  | ● | ● |  |
|  |  | TW13-031e | *B.* sp. | TW |  |  |  |  |  |  |  | ● | ● |  |
|  |  | TW13-031f | *B.* sp. | TW |  |  |  |  |  |  |  | ● | ● |  |
|  | TW13-033 |  |  | TW | Fengbin, Hualian, Taiwan | h23 |  |  | ○ |  |  |  |  | 2013.03.20 |
|  | TW13-034 |  |  | TW | Fengbin, Hualian, Taiwan | h23 |  |  | ○ |  |  |  |  | 2013.03.20 |
|  | TW13-035 |  |  | TW | Fengbin, Hualian, Taiwan | h23 |  |  | ○ |  |  |  |  | 2013.03.20 |
|  | TW13-036 |  |  | TW | Fengbin, Hualian, Taiwan | h23 |  |  | ○ |  |  |  |  | 2013.03.20 |
|  | TW13-037 |  |  | TW | Fengbin, Hualian, Taiwan | h23 |  |  | ○ |  |  |  |  | 2013.03.20 |
|  | TW13-039 |  |  | TW | Fengbin, Hualian, Taiwan | h23 |  |  | ○ |  |  |  |  | 2013.03.20 |
|  | TW13-040 |  |  | TW | Fengbin, Hualian, Taiwan | h23 |  |  | ○ |  |  |  |  | 2013.03.20 |
|  | TW13-041 |  |  | TW | Fengbin, Hualian, Taiwan | h23 |  |  | ○ |  |  |  |  | 2013.03.20 |
|  | TW13-042 |  |  | TW | Fengbin, Hualian, Taiwan | h23 |  |  | ○ |  |  |  |  | 2013.03.20 |
|  | TW13-043 |  |  | TW | Fengbin, Hualian, Taiwan | h23 |  |  | ○ |  |  |  |  | 2013.03.20 |
|  | TW14-007 | TW14-007-A | *B.* sp. | TW | Fengbin, Hualian, Taiwan |  |  |  |  |  | ○ | ○ | ○ | 2014.03.21 |
|  |  | TW14-007-B | *B.* sp. | TW |  |  |  |  |  |  | ○ |  | ○ |  |
|  |  | TW14-007-d | *B.* sp. | TW |  |  |  |  |  |  |  |  | ○ |  |
|  | TW14-010 | TW14-010 | *B.* sp. | TW | Fengbin, Hualian, Taiwan |  |  |  |  |  |  |  | ○ | 2014.03.21 |
|  | TW14-018 | TW14-018-A | *B. yeni* | TW | Kenting, Pingdong, Taiwan |  |  |  |  |  |  | ○ | ○ | 2014.03.23 |
|  |  | TW14-018-B | *B. yeni* | TW |  |  |  |  |  |  |  |  | ○ |  |
|  |  | TW14-018-D | *B. yeni* | TW |  |  |  |  |  |  |  |  | ○ |  |
|  | TW16-217 |  |  | TW | Guishandao, Toucheng, Yilan, Taiwan | h24 |  |  | ○ |  |  |  |  | 2016.08.12 |
|  | TW16-219 |  |  | TW | Guishandao, Toucheng, Yilan, Taiwan | h24 |  |  | ○ |  |  |  |  | 2016.08.12 |
|  | TW16-220 |  |  | TW | Guishandao, Toucheng, Yilan, Taiwan | h24 |  |  | ○ |  |  |  |  | 2016.08.12 |
|  | TW18-130 | TW18-130A | *B. yeni* | TW | Ludao, Taidong, Taiwan |  |  |  | ○ |  | ○ | ○ | ○ | 2018.08.30 |
|  |  | TW18-130B | *B. yeni* | TW |  |  |  |  |  |  | ○ | ○ | ○ |  |
|  | TW18-131 | TW18-131A | *B. yeni* | TW | Ludao, Taidong, Taiwan |  |  |  | ○ |  |  | ○ | ○ | 2018.08.30 |
|  |  | TW18-131B | *B. yeni* | TW |  |  |  |  |  |  |  | ○ | ○ |  |
|  | TW18-134 | TW18-134A | *B. yeni* | TW | Ludao, Taidong, Taiwan |  |  |  | ○ |  |  | ○ | ○ | 2018.08.30 |
|  | TW18-135 | TW18-135A | *B. yeni* | TW | Ludao, Taidong, Taiwan |  |  |  | ○ |  |  | ○ | ○ | 2018.08.30 |
|  |  | TW18-135B | *B. yeni* | TW |  |  |  |  |  |  |  | ○ | ○ |  |
|  |  | TW18-135C | *B. yeni* | TW |  |  |  |  |  |  |  | ○ | ○ |  |
|  |  | TW18-135D | *B. yeni* | TW |  |  |  |  |  |  |  | ○ | ○ |  |
|  |  | TW18-287 | *B. yeni* | TW | Liangshan Waterfall, Pingtung, Taiwan |  |  |  |  |  |  | ○ | ○ |  |
| Sub-total | 34 | 24 |  | TW |  | 26 |  | 5 | 30 | 1 | 4 | 19 | 34 |  |
|  | TW11-155 |  |  | LY | Yeyou, Lanyu, Taiwan | h23 |  |  | ● |  |  |  |  | 2011.09.12 |
|  | TW11-156 |  |  | LY | Yeyou, Lanyu, Taiwan | h23 |  |  | ● |  |  |  |  | 2011.09.12 |
|  | TW11-157 |  |  | LY | Yeyou, Lanyu, Taiwan | h23 |  |  | ● |  |  |  |  | 2011.09.12 |
|  | TW11-158 |  |  | LY | Yeyou, Lanyu, Taiwan | h23 |  |  | ● |  |  |  |  | 2011.09.12 |
|  | TW11-159 |  |  | LY | Yeyou, Lanyu, Taiwan | h23 |  |  | ● |  |  |  |  | 2011.09.12 |
|  | TW11-160 |  |  | LY | Yeyou, Lanyu, Taiwan | h23 |  | ○ | ● |  |  |  |  | 2011.09.12 |
|  | TW11-163 |  |  | LY | Yeyou, Lanyu, Taiwan | h23 |  | ○ | ● |  |  |  |  | 2011.09.12 |
|  | TW11-164 | TW11-164-2 | *B. yeni* | LY | Yeyou, Lanyu, Taiwan | h23 |  | ○ | ● | ○ |  |  | ● | 2011.09.12 |
|  |  | TW11-164-3 | *B. yeni* | LY |  |  |  |  |  |  |  | ● | ● |  |
|  | TW11-165 | TW11-165-1 | *B. yeni* | LY | Xiaotienchih, Lanyu, Taiwan | h23 |  | ○ | ● | ○ |  |  | ● | 2011.09.12 |
|  |  | TW11-165-4 | *B. yeni* | LY |  |  |  |  |  |  |  |  | ● |  |
|  | TW11-173 | TW11-173-1 | *B. yeni* | LY | Yeyou, Lanyu, Taiwan | h23 |  | ○ | ● | ○ |  | ● | ● | 2011.09.13 |
| Sub-total | 10 | 5 |  | LY |  | 10 |  | 5 | 10 | 3 |  | 2 | 5 |  |
| *F. abelii* | C18-019 | C18-019A | *B. silvestriana* | GD | Conghua, Guangzhou, Guangdong, China |  | ○ | ○ |  | ○ | ○ | ○ |  | 2018.04.24 |
|  |  | C18-019B | *B. silvestriana* | GD |  |  |  |  |  |  | ○ | ○ |  |  |
|  |  | C18-019C | *B. silvestriana* | GD |  |  |  |  |  |  |  | ○ |  |  |
|  |  | C18-019D | *B. silvestriana* | GD |  |  |  |  |  |  |  | ○ |  |  |
|  |  | C18-019E | *B. silvestriana* | GD |  |  |  |  |  |  |  | ○ |  |  |
|  | C18-027 | C18-027A | *B. silvestriana* | GD | Conghua, Guangzhou, Guangdong, China |  | ○ | ○ |  | ○ | ○ | ○ |  | 2018.04.24 |
|  |  | C18-027B | *B. silvestriana* | GD |  |  |  |  |  |  | ○ | ○ |  |  |
|  |  | C18-027C | *B. silvestriana* | GD |  |  |  |  |  |  |  | ○ |  |  |
|  |  | C18-027D | *B. silvestriana* | GD |  |  |  |  |  |  |  | ○ |  |  |
| Sub-total | 2 | 9 |  | GD |  |  | 2 | 2 |  | 2 | 4 | 9 |  |  |
| *F. pyriformis* | C18-035 | C18-035A | *B. silvestriana* | GD | Zhaoqing, Guangdong, China |  |  | ○ |  | ○ | ○ | ○ |  | 2018.04.25 |
|  |  | C18-035B | *B. silvestriana* | GD |  |  |  |  |  |  | ○ | ○ |  |  |
|  | C18-036 |  |  | GD | Zhaoqing, Guangdong, China |  |  |  |  |  |  |  |  | 2018.04.25 |
|  | C18-037 |  |  | GD | Zhaoqing, Guangdong, China |  | ○ |  |  |  |  |  |  | 2018.04.25 |
|  | C18-038 | C18-038A | *B. silvestriana* | GD | Zhaoqing, Guangdong, China |  | ○ | ○ |  |  |  | ○ |  | 2018.04.25 |
|  |  | C18-038B | *B. silvestriana* | GD |  |  |  |  |  |  |  | ○ |  |  |
|  | C18-040 | C18-040mA | *B. silvestriana* | GD | Zhaoqing, Guangdong, China |  | ○○* | ○ |  |  |  | ○ |  | 2018.04.25 |
|  |  | C18-040mB | *B. silvestriana* | GD |  |  |  |  |  |  |  | ○ |  |  |
|  | C18-041 |  |  | GD | Zhaoqing, Guangdong, China |  | ○ |  |  |  |  |  |  | 2018.04.25 |
|  | C18-042 | C18-042mA | *B. silvestriana* | GD | Zhaoqing, Guangdong, China |  | ○ | ○ |  |  |  | ○ |  | 2018.04.25 |
|  | C18-043 | C18-043A | *B. silvestriana* | GD | Zhaoqing, Guangdong, China |  |  |  |  |  |  | ○ |  | 2018.04.25 |
|  |  | C18-043B | *B. silvestriana* | GD |  |  |  |  |  |  |  | ○ |  |  |
|  | C18-046 | C18-046A | *B. silvestriana* | GD | Zhaoqing, Guangdong, China |  |  |  |  | ○ |  | ○ |  | 2018.04.25 |
|  |  | C18-046B | *B. silvestriana* | GD |  |  |  |  |  |  |  | ○ |  |  |
|  |  | C18-046C | *B. silvestriana* | GD |  |  |  |  |  |  |  | ○ |  |  |
|  |  | C18-046D | *B. silvestriana* | GD |  |  |  |  |  |  |  | ○ |  |  |
|  | C18-047 | C18-047 | *B. silvestriana* | GD | Zhaoqing, Guangdong, China |  |  |  |  |  |  | ○ |  | 2018.04.25 |
|  | C18-050 | C18-050A | *B. silvestriana* | HK | Mt. Taimo-san, Hong Kong |  |  | ○ |  | ○ | ○ | ○ |  | 2018.04.28 |
|  | C18-051 | C18-051A | *B. silvestriana* | HK | Mt. Taimo-san, Hong Kong |  |  | ○ |  | ○ | ○ | ○ |  | 2018.04.28 |
|  | C18-052 |  |  | HK | Mt. Taimo-san, Hong Kong |  |  | ○ |  |  |  |  |  | 2018.04.28 |
|  | C18-053 |  |  | HK | Mt. Taimo-san, Hong Kong |  |  | ○ |  |  |  |  |  | 2018.04.28 |
| Sub-total | 14 | 16 |  | GD, HK |  |  | 6 | 8 |  | 4 | 4 | 16 |  |  |
| *F. variolosa* | C17-006 | C17-006A | *B. silvestriana* | FJ | Jiaocheng, Ningde, Fujian, China |  |  |  |  | ○ | ○ | ○ |  | 2017.08.12 |
|  |  | C17-006B | *B. silvestriana* | FJ |  |  |  |  |  | ○ | ○ | ○ |  |  |
|  |  | C17-006C | *B. silvestriana* | FJ |  |  |  |  |  | ○ |  | ○ |  |  |
|  | C18-033 | C18-033 | *B. silvestriana* | GD | Conghua, Guangzhou, Guangdong, China |  |  |  |  | ○ | ○ | ○ |  | 2018.04.24 |
|  | C18-064 |  |  | HK | Pat Sin Leng Country Park, Hong Kong |  |  |  |  |  |  |  |  | 2018.04.28 |
|  | C18-065 |  |  | HK | Pat Sin Leng Country Park, Hong Kong |  |  |  |  |  |  |  |  | 2018.04.28 |
|  | C18-069 | C18-069A | *B. silvestriana* | HK | Pat Sin Leng Country Park, Hong Kong |  |  | ○ |  | ○ | ○ | ○ |  | 2018.04.28 |
|  |  | C18-069B | *B. silvestriana* | HK |  |  |  |  |  |  | ○ | ○ |  |  |
|  |  | C18-069C | *B. silvestriana* | HK |  |  |  |  |  |  |  | ○ |  |  |
|  |  | C18-069D | *B. silvestriana* | HK |  |  |  |  |  |  |  | ○ |  |  |
|  |  | C18-069E | *B. silvestriana* | HK |  |  |  |  |  |  |  | ○ |  |  |
|  | C18-070 | C18-070A | *B. silvestriana* | HK | Sai kung East Country Park, Hong Kong |  |  | ○ |  | ○ |  | ○ |  | 2018.04.29 |
|  |  | C18-070B | *B. silvestriana* | HK |  |  |  |  |  |  |  | ○ |  |  |
|  |  | C18-070C | *B. silvestriana* | HK |  |  |  |  |  |  |  | ○ |  |  |
|  |  | C18-070D | *B. silvestriana* | HK |  |  |  |  |  |  |  | ○ |  |  |
|  | C18-089 |  |  | HK | Mt. Kowloon, Hong Kong |  |  | ○ |  |  |  |  |  | 2018.04.30 |
|  | C18-090 |  |  | HK | Mt. Kowloon, Hong Kong |  |  | ○ |  |  |  |  |  | 2018.04.30 |
|  | C18-092 | C18-092A | *B. silvestriana* | HK | Mt. Kowloon, Hong Kong |  |  | ○ |  |  |  | ○ |  | 2018.04.30 |
|  |  | C18-092C | *B. silvestriana* | HK |  |  |  |  |  |  |  | ○ |  |  |
|  |  | C18-092D | *B. silvestriana* | HK |  |  |  |  |  |  |  | ○ |  |  |
|  |  | C18-092E | *B. silvestriana* | HK |  |  |  |  |  |  |  | ○ |  |  |
| Sub-total | 9 | 17 |  | FJ, GD, HK |  |  |  | 5 |  | 6 | 5 | 17 |  |  |
| outgroup |  |  |  |  |  |  |  |  |  |  |  |  |  |  |
| *F. nipponica* | J17-689 |  |  |  | Shimantoshi, Kochi, Japan |  |  | ○ |  |  |  |  |  | 2017.09.06 |
|  | J17-710 |  |  |  | Ibaraki, Osaka, Japan |  |  | ○ |  |  |  |  |  | 2017.09.13 |
|  | J18-386 |  |  |  | Zamamijima, Okinawa, Japan |  |  | ○ |  |  |  |  |  | 2018.05.03 |
| *F. thunbergii* | OK17-060 |  |  |  | Kunigami, Okinawa, Japan |  |  | ○ |  |  |  |  |  | 2017.07.23 |
|  | OK17-087 |  |  |  | Nago, Okinawa, Japan |  |  | ○ |  |  |  |  |  | 2017.07.25 |
|  | J18-127 |  |  |  | Amami-oshima, Kagoshima, Japan |  |  | ○ |  |  |  |  |  | 2018.04.13 |
| *F. ampelas* |  | su077 | *Kradibia sumatrana* |  | Inoda, Ishigaki, Okinawa, Japan |  |  |  |  |  |  | ○ |  | 2002.06.03 |
|  |  | su061 | *K. sumatrana* |  | Funaura, Iriomote, Okinawa, Japan |  |  |  |  | ● |  |  |  | 2002.05.29 |
| *F. irisana* |  | su083 | *K. commuta* |  | Ohtake, Ishigaki, Okinawa, Japan |  |  |  |  | ● |  | ○ |  | 2002.06.04 |
| *F. virgata* |  | J17-285C | *Liporrhopalum philippinensis* |  | Ishigaki, Okinawa, Japan |  |  |  |  |  |  | ○ |  | 2017.04.28 |
|  |  | su014 | *L. philippinensis* |  | Hatomajima, Taketomi-cho, Okinawa, Japan |  |  |  |  | ● |  |  |  | 2002.02.23 |
| *F. benguetensis* |  | J16-009A | *Ceratosolen cornutus* |  | Ishigaki, Okinawa, Japan |  |  |  |  |  |  | ○ |  | 2016.06.02 |
|  |  | su005 | *C. cornutus* |  | Iriomote, Okinawa, Japan |  |  |  |  | ● |  |  |  | 2002.02.21 |
| *F. septica* |  | J12-044-1 | *Ceratosolen* sp. |  | Ishigaki, Okinawa, Japan |  |  |  |  |  |  | ○ |  | 2012.05.05 |
|  |  | su003 | *Ceratosolen* sp. |  | Iriomote, Okinawa, Japan |  |  |  |  | ● |  |  |  | 2002.02.21 |
| *F. subpisocarpa* |  | J17-777A | *Platyscapa* sp. |  | Ishigaki, Okinawa, Japan |  |  |  |  |  |  | ○ |  | 2017.11.06 |
|  |  | su057 | *Platyscapa* sp. |  | Asato, Gushikami, Okinawa, Japan |  |  |  |  | ● |  |  |  | 2002.05.26 |
| *F. microcarpa* |  | J17-069A | *Eupristina verticillata* |  | Ishigaki, Okinawa, Japan |  |  |  |  |  |  | ○ |  | 2017.04.22 |
|  |  | su013 | *E. verticillata* |  | Hatomajima, Taketomi-cho, Okinawa, Japan |  |  |  |  | ● |  |  |  | 2002.02.23 |
| *F. crocata* |  | MX08-055 | *Pegoscapus* sp. |  | Axochiapan-Tepalcingo, South Morelos, Mexico |  |  |  |  | ○ |  | ○ |  | 2008.10.03 |
| *F. cotinifolia* |  | MX08-077 | *P. karussii* |  | Lazaro Cardenas, South Michoacan, Mexico |  |  |  |  | ○ |  | ○ |  | 2008.10.06 |
|  |  | MX08-101 | *P. karussii* |  | Santiago Tangamandapio, Michoacan, Mexico |  |  |  |  | ○ |  | ○ |  | 2008.10.10 |
| *F. punctata* |  | AF200414, AY616578 | *Wiebesia punctatae* |  |  |  |  |  |  | ● |  | ● |  |  |
| *F. ischnopoda* |  | Y033 | *Blastophaga* sp. |  | Xishuangbanna Botanical Garden, Yunna, China |  |  |  |  | ○ |  | ○ |  | 2006.07.27 |
| Sub-total | 6 | 18 |  |  |  |  |  | 6 |  | 12 |  | 12 |  |  |
| Total | 528 | 411 |  |  |  | 418 | 21 | 60 | 447 | 134 | 37 | 329 | 263 |  |
| Plastid sequence data are shown with haplotype numbers (blue numbers: analyzed previously). For *COI*-*COII* data, cell color shows the same sequences in each population. ○, analyzed in this study; ●, analyzed in previous studies^16,24,36^; *, ITS sequences were also determined from pollen carried by foundress wasps collected from inside syconia (Ref. Supplementary Figure 3). | | | | | | | | | | | | | | |
| KS, Kansai; NK, N Kyushu; SK, S Kyushu; AM, Amami-Oshima Island; OK, Okinawa Island; IS, Ishigaki Island; IR, Iriomote Island; YN, Yonaguni Island; TW, Taiwan; LY, Lanyu Island; MT, Matsu Islands; FJ, Fujian; GD, Guangdong; HK, Hong Kong. | | | | | | | | | | | | | | |
