# Supplementary material for: Pollinator sharing, copollination, and speciation by host shifting among six closely related dioecious fig species
Source: Commun Biol. 2022 Apr 8;5:284. doi: 10.1038/s42003-022-03223-0 (PMC8993897; doi:10.1038/s42003-022-03223-0)
Supplement: Supplementary file 5 — Reporting Summary [file 42003_2022_3223_MOESM5_ESM.pdf]

nature research

Corresponding author(s): Zhi-Hui Su
Last updated by author(s): Jan 28, 2022

Reporting Summary

Nature Research wishes to improve the reproducibility of the work that we publish. This form provides structure for consistency and transparency in reporting. For further information on Nature Research policies, see our Editorial Policies and the Editorial Policy Checklist.

Statistics

For all statistical analyses, confirm that the following items are present in the figure legend, table legend, main text, or Methods section.

n/a	Confirmed	
☒	☐	The exact sample size (*n*) for each experimental group/condition, given as a discrete number and unit of measurement
☒	☐	A statement on whether measurements were taken from distinct samples or whether the same sample was measured repeatedly
☒	☐	The statistical test(s) used AND whether they are one- or two-sided<br>*Only common tests should be described solely by name; describe more complex techniques in the Methods section.*
☒	☐	A description of all covariates tested
☒	☐	A description of any assumptions or corrections, such as tests of normality and adjustment for multiple comparisons
☒	☐	A full description of the statistical parameters including central tendency (e.g. means) or other basic estimates (e.g. regression coefficient) AND variation (e.g. standard deviation) or associated estimates of uncertainty (e.g. confidence intervals)
☒	☐	For null hypothesis testing, the test statistic (e.g. *F*, *t*, *r*) with confidence intervals, effect sizes, degrees of freedom and *P* value noted<br>*Give P values as exact values whenever suitable.*
☒	☐	For Bayesian analysis, information on the choice of priors and Markov chain Monte Carlo settings
☒	☐	For hierarchical and complex designs, identification of the appropriate level for tests and full reporting of outcomes
☒	☐	Estimates of effect sizes (e.g. Cohen's *d*, Pearson's *r*), indicating how they were calculated

Our web collection on statistics for biologists contains articles on many of the points above.

Software and code

Policy information about availability of computer code

Data collection	The softwares used for data collection in this study are described in the manuscript.
Data analysis	The softwares used for data analysis in this study are described in the manuscript.

For manuscripts utilizing custom algorithms or software that are central to the research but not yet described in published literature, software must be made available to editors and reviewers. We strongly encourage code deposition in a community repository (e.g. GitHub). See the Nature Research guidelines for submitting code & software for further information.

Data

Policy information about availability of data

All manuscripts must include a data availability statement. This statement should provide the following information, where applicable:

- Accession codes, unique identifiers, or web links for publicly available datasets
- A list of figures that have associated raw data
- A description of any restrictions on data availability

The sequence data were deposited in the DDBJ/EMBL/GenBank under the following accession numbers: DRR315461-DRR315520 for MIG-seq, LC626904-LC627035 and LC647291-LC647338 for plastid DNA, LC626702-LC626722 for ITS, LC647577-LC647632 for ITS1, LC627753-LC627757 and LC647276-LC647290 for 28S rRNA, LC627616-LC627750 and LC647633-LC647648 for COI-COII. The DNA sequence alignments, the calibrated phylogenetic tree, and SSR data are available at https://doi:10.5061/dryad.3n5tb2rj9.

Field-specific reporting

Please select the one below that is the best fit for your research. If you are not sure, read the appropriate sections before making your selection.

☐ Life sciences ☐ Behavioural & social sciences ☒ Ecological, evolutionary & environmental sciences

For a reference copy of the document with all sections, see nature.com/documents/nr-reporting-summary-flat.pdf

Ecological, evolutionary & environmental sciences study design

All studies must disclose on these points even when the disclosure is negative.

Study description	This study is to investigate the phylogenetic relationships and population genetic structures of six closely related fig species and their pollinators from southern China and Taiwan-Ryukyu islands.
Research sample	The sample information was described in the manuscript.
Sampling strategy	Sampling strategy was described in the Methods of the manuscript.
Data collection	n/a
Timing and spatial scale	n/a
Data exclusions	n/a
Reproducibility	n/a
Randomization	n/a
Blinding	n/a

Did the study involve field work? ☒ Yes ☐ No

Field work, collection and transport

Field conditions	Only sampling was conducted in field. No experiments were conducted in field.
Location	The location of the sampling was shown in Fig. 1.
Access & import/export	The samples were collected according to the required regulations. The samples collected in field were stored in ethanol solution and brought back to the laboratory.
Disturbance	n/a

Reporting for specific materials, systems and methods

We require information from authors about some types of materials, experimental systems and methods used in many studies. Here, indicate whether each material, system or method listed is relevant to your study. If you are not sure if a list item applies to your research, read the appropriate section before selecting a response.

Materials & experimental systems

n/a	Involved in the study	
☒	☐	Antibodies
☒	☐	Eukaryotic cell lines
☒	☐	Palaeontology and archaeology
☐	☒	Animals and other organisms
☒	☐	Human research participants
☒	☐	Clinical data
☒	☐	Dual use research of concern

Methods

n/a	Involved in the study	
☒	☐	ChIP-seq
☒	☐	Flow cytometry
☒	☐	MRI-based neuroimaging

Animals and other organisms

Policy information about studies involving animals; ARRIVE guidelines recommended for reporting animal research

Laboratory animals	No laboratory animals were used in this study.
Wild animals	The sample information of fig wasps (insects) used in this study was provided in the manuscript.
Field-collected samples	All samples used in this study were collected from the field. Sample information was provided in the manuscript.
Ethics oversight	This study was conducted according to the guidance provided by JT Biohistory Research Hall.

Note that full information on the approval of the study protocol must also be provided in the manuscript.
